# Supplementary material for: Molecular basis of antibiotic self-resistance in a bee larvae pathogen
Source: Nat Commun. 2022 Apr 29;13:2349. doi: 10.1038/s41467-022-29829-w (PMC9054821; doi:10.1038/s41467-022-29829-w)
Supplement: Supplementary file 1 — Supplementary Information [file 41467_2022_29829_MOESM1_ESM.pdf]

Supplementary Information

Molecular Basis of Antibiotic Self-Resistance in a Bee Larvae Pathogen

Tam Dang[1], Bernhard Loll[2], Sebastian Müller[1], Ranko Skobalj[1], Julia Ebeling[3], Timur Bulatov[1], Sebastian Gensel[1], Josefine Göbel[3], Markus C. Wahl[2,4], Elke Genersch[3,5], Andi Mainz[1] and Roderich D. Süssmuth[1,*]

[1] Institut für Chemie, Technische Universität Berlin, Berlin, Germany

[2] Institut für Chemie und Biochemie, Strukturbiochemie, Freie Universität Berlin, Berlin, Germany

[3] Institute for Bee Research, Department of Molecular Microbiology and Bee Diseases, Hohen Neuendorf, Germany

[4] Macromolecular Crystallography, Helmholtz Zentrum Berlin für Materialien und Energie, Berlin, Germany

[5] Institut für Mikrobiologie und Tierseuchen, Fachbereich Veterinärmedizin, Freie Universität Berlin, Berlin, Germany

* Corresponding author

Table of Contents

Supplementary Figures 2
Supplementary Tables 20
References 24

Supplementary Figures

Supplementary Figure 1. Protein purification of PamZ including TEV cleavage.

Supplementary Figure 2. Deconvoluted MS^1 spectra of native paenilamicin variants observed in the supernatant of *P. larvae* ATCC 9545 (ERIC I) and DSM 25430 (ERIC II) after purification with Amberlite XAD16.

Supplementary Figure 3. MS^1 spectra of *in vitro* activation assay including paenilamicin A1, acetyl-CoA and PamZ.

Supplementary Figure 4. MS^1 spectra of *in vitro* activation assay including paenilamicin A2, acetyl-CoA and PamZ.

Supplementary Figure 5. MS^1 spectra of *in vitro* activation assay including paenilamicin B1, acetyl-CoA and PamZ.

Supplementary Figure 6. MS^1 spectra of *in vitro* activation assay including paenilamicin B2, acetyl-CoA and PamZ.

Supplementary Figure 7. MS^1 spectra of *in vitro* time-dependent activation assay including paenilamicin B2 (synthetic), acetyl-CoA and PamZ.

Supplementary Figure 8. MS^2 spectra of paenilamicin A1 isolated from *P. larvae* DSM 25430 (ERIC II).

Supplementary Figure 9. MS^2 spectra of paenilamicin A2 isolated from *P. larvae* ATCC 9545 (ERIC I).

Supplementary Figure 10. MS^2 spectra of paenilamicin B1 isolated from *P. larvae* DSM 25430 (ERIC II).

Supplementary Figure 11. MS^2 spectra of paenilamicin B2 isolated from *P. larvae* ATCC 9545 (ERIC I).

Supplementary Figure 12. MS^2 spectra of paenilamicin B2 (synthetic).

Supplementary Figure 13. MS^2 spectrum of *N*-acetylpaenilamicin A1 converted *in vitro* by PamZ.

Supplementary Figure 14. MS^2 spectra of *N*-acetylpaenilamicin A2 converted *in vitro* by PamZ.

Supplementary Figure 15. MS^2 spectra of *N*-acetylpaenilamicin B1 converted *in vitro* by PamZ.

Supplementary Figure 16. MS^2 spectra of *N*-acetylpaenilamicin B2 converted *in vitro* by PamZ.

Supplementary Figure 17. MS^2 spectra of *N*-acetylpaenilamicin B2 (synthetic) converted *in vitro* by PamZ.

Supplementary Figure 18. MS^2 fragmentation of different paenilamicin and *N*-acetylpaenilamicin variants to determine regioselective acetylation.

Supplementary Figure 19. MS^1 spectra of *N*-acetylpaenilamicin A1, B1 and B2 isolated from *P. larvae*.

Supplementary Figure 20. MS^2 spectra of *N*-acetylpaenilamicin B2 isolated from *P. larvae* DSM 25430 (ERIC II).

Supplementary Figure 21. MS^2 spectra of *N*-acetylpaenilamicin B1 isolated from *P. larvae* DSM 25430 (ERIC II).

Supplementary Figure 22. MS^2 spectra of *N*-acetylpaenilamicin A1 isolated from *P. larvae* ATCC 9545 (ERIC I).

Supplementary Figure 23. Stereoselective *N*-acetylation of PamZ.

Supplementary Figure 24. Multiple sequence alignment of Gcn5-related *N*-acetyltransferases of PamZ (paenilamicin), ZmaR (zwittermicin) and EdeQ (edeine).

Supplementary Figure 25. Polder electron density map of acetyl-CoA.

Supplementary Figure 26. Structural comparison of PamZ and AACs.

Supplementary Figure 27. Pairwise structural alignment between NTD and CTD of PamZ.

Supplementary Figure 28. Size exclusion chromatogram and calibration curve of PamZ including calibration standards detected at 280 nm.

Supplementary Figure 29. Biosynthetic gene cluster and *N*-acetylation of paenilamicin, zwittermicin A, edeine and amicoumacin A.

Supplementary Figure 30. Determination of successful intron insertion into the *pamZ* gene of *P. larvae* DSM 25430.

Supplementary Figure 31. ^{1}H-NMR spectrum of paenilamicin A2 isolated from *P. larvae* ATCC 9545.

Supplementary Figure 32. ^{1}H-NMR spectrum of paenilamicin B2 isolated from *P. larvae* ATCC 9545.

Supplementary Figure 33. ^{1}H-NMR spectrum of paenilamicin A1 isolated from *P. larvae* DSM 25430.

Supplementary Figure 34. ^{1}H-NMR spectrum of paenilamicin B1 isolated from *P. larvae* DSM 25430.

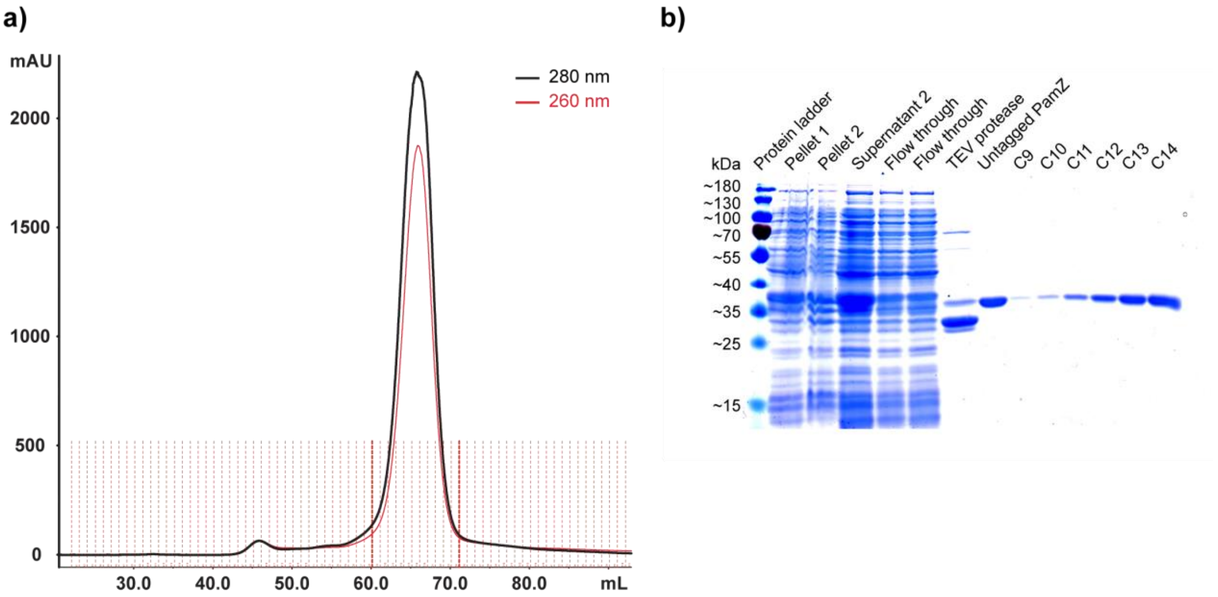

Supplementary Figure 1. Protein purification of PamZ including TEV cleavage. a) Elution profile of size exclusion chromatography after TEV cleavage observed at 280 nm. **b)** SDS-PAGE of PamZ after protein purification. Fractions C9-C14 were obtained from size exclusion chromatography and concentrated for further applications. The purification of PamZ including the SDS-PAGE were repeated at least three times independently. Source data are provided as a Source Data file.

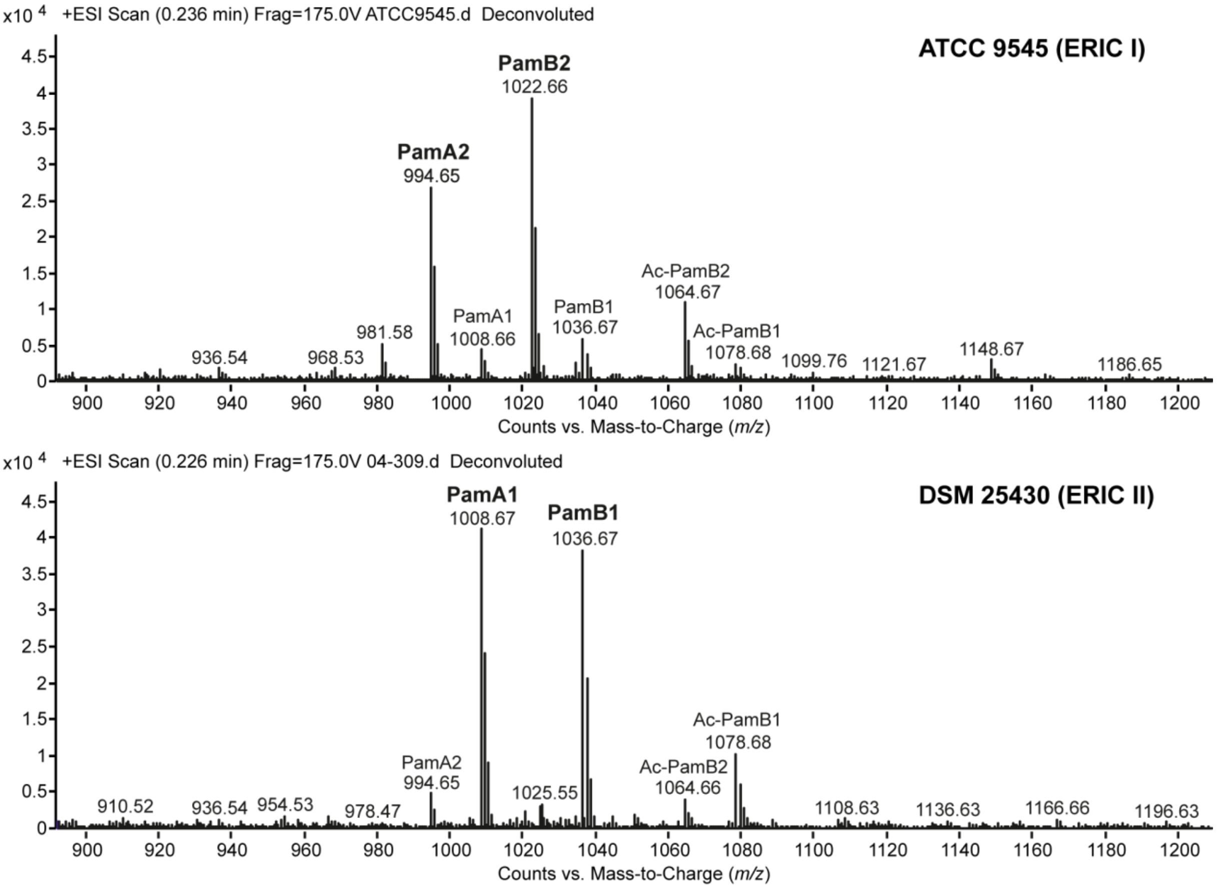

Supplementary Figure 2. Deconvoluted MS^1 spectra of native paenilamicin variants observed in the supernatant of *P. larvae* ATCC 9545 (ERIC I) and DSM 25430 (ERIC II) after purification with Amberlite XAD16.

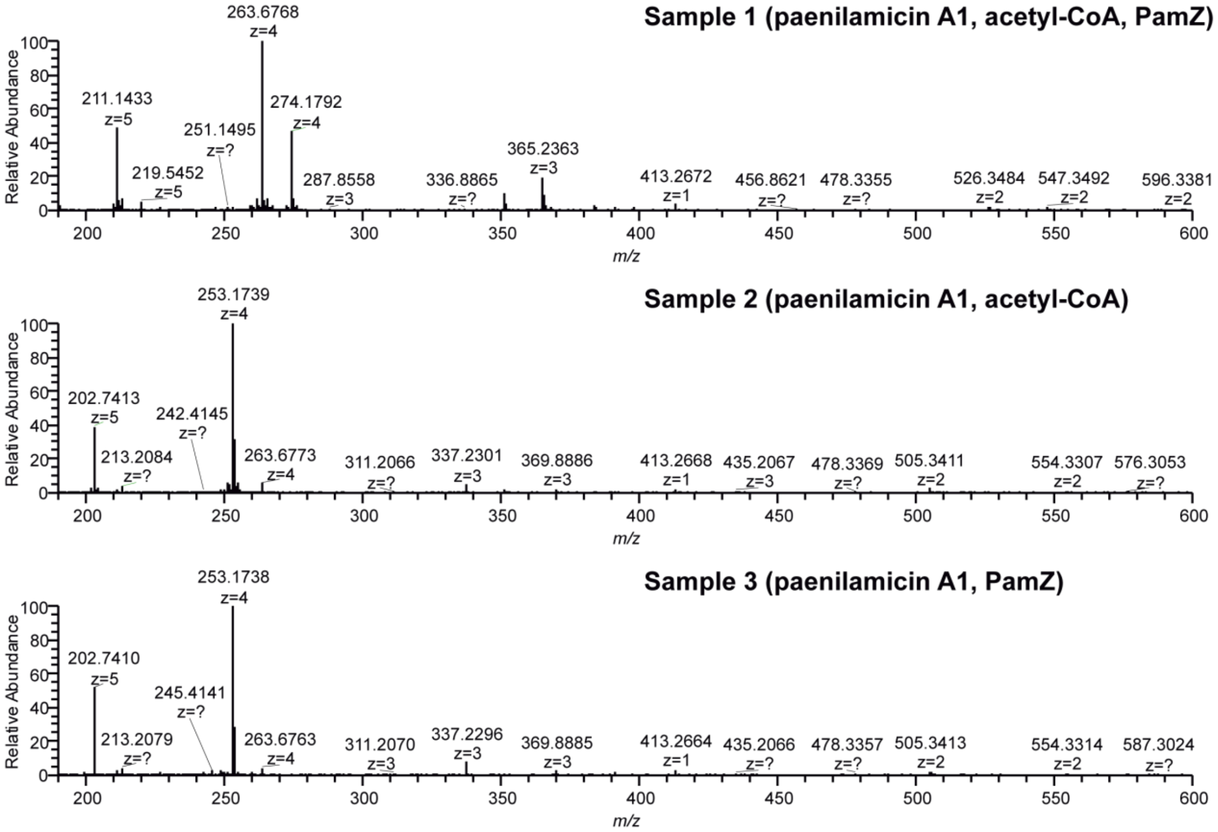

Supplementary Figure 3. MS[1] spectra of *in vitro* activation assay including paenilamicin A1, acetyl-CoA and PamZ.

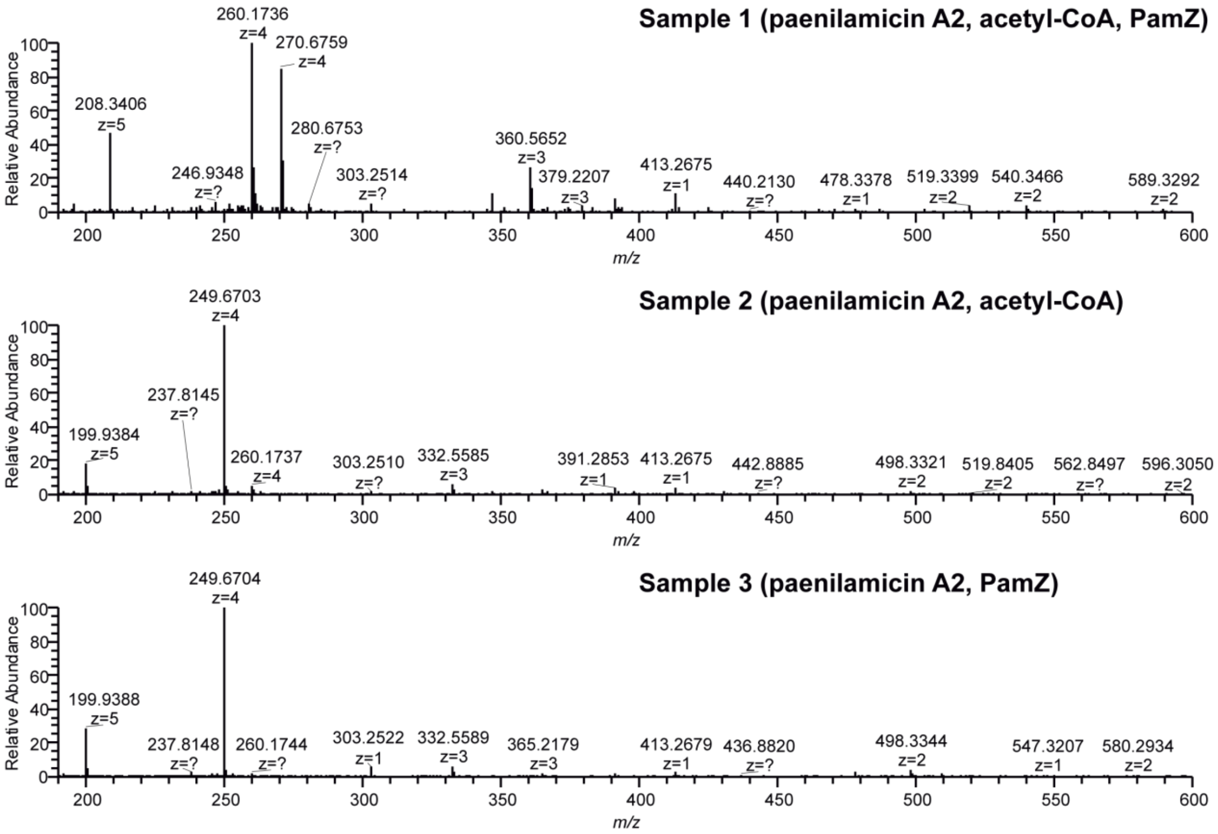

Supplementary Figure 4. MS[1] spectra of *in vitro* activation assay including paenilamicin A2, acetyl-CoA and PamZ.

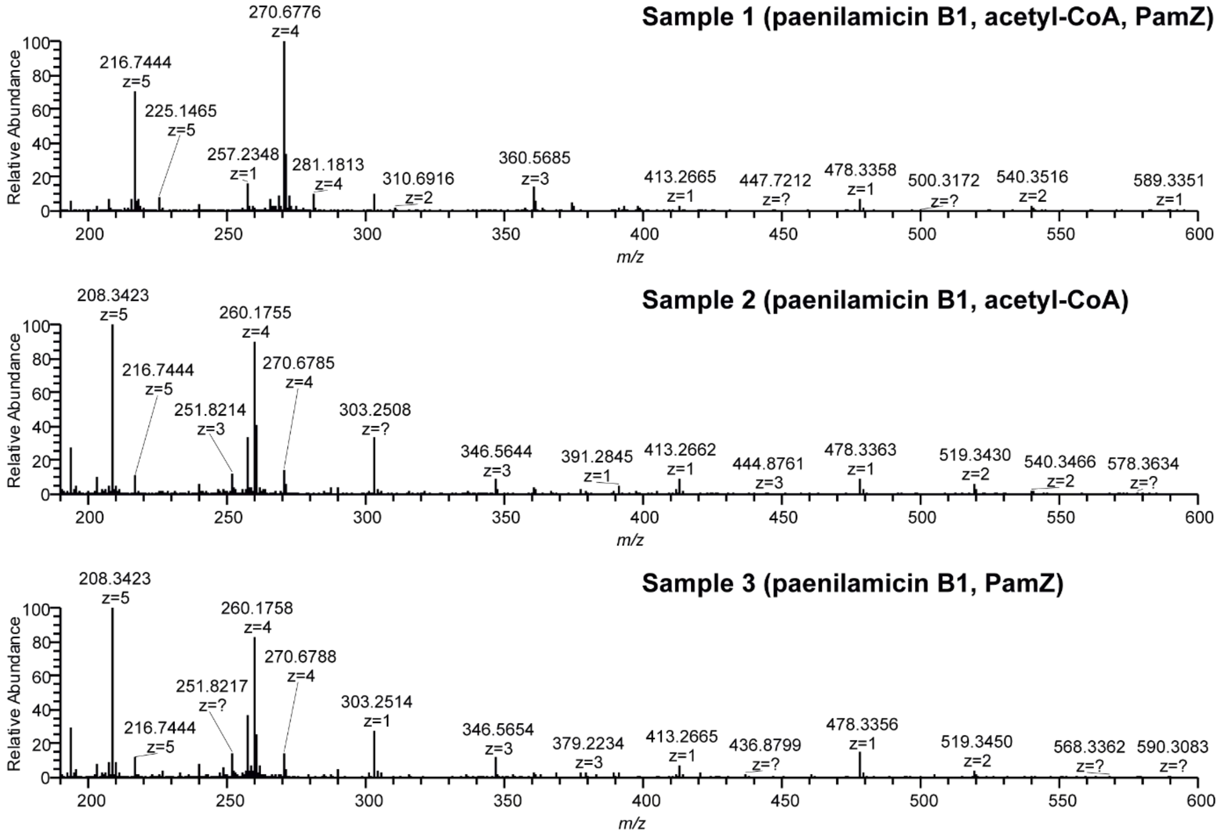

Supplementary Figure 5. MS[1] spectra of *in vitro* activation assay including paenilamicin B1, acetyl-CoA and PamZ.

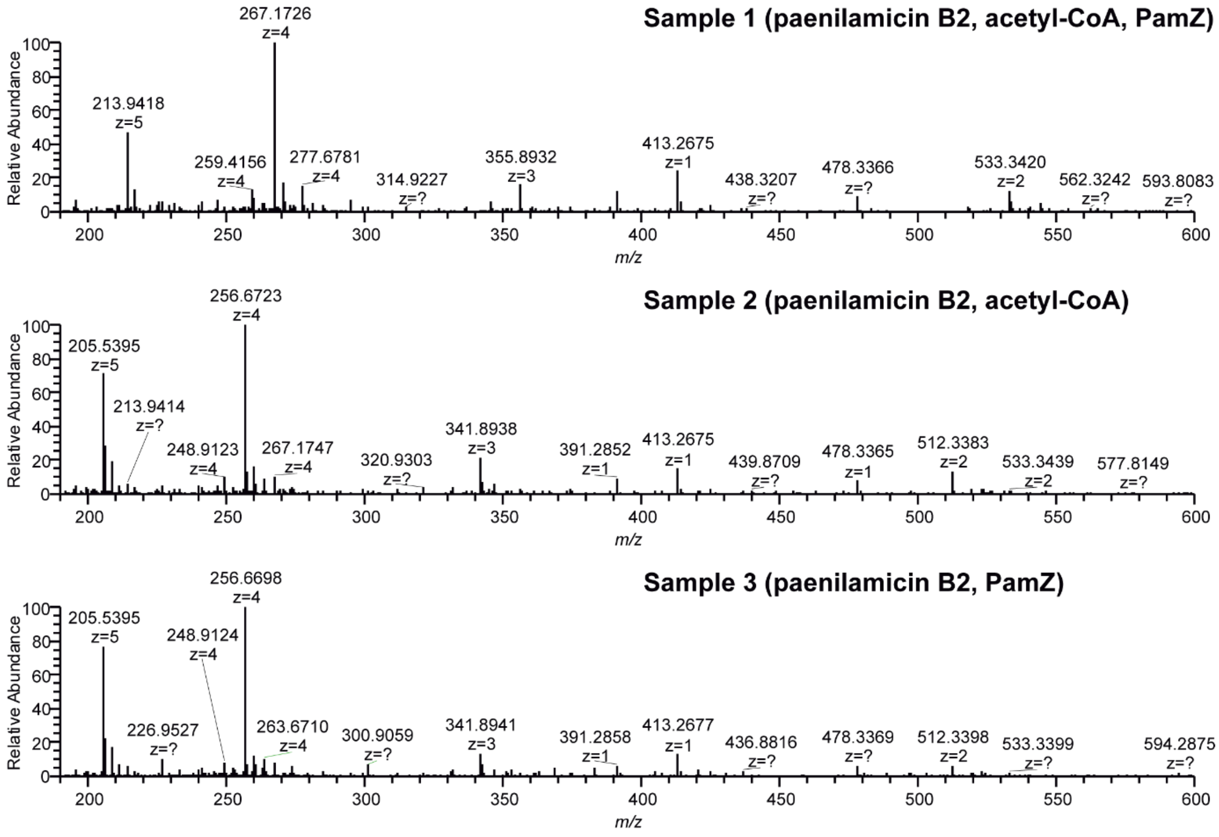

Supplementary Figure 6. MS[1] spectra of *in vitro* activation assay including paenilamicin B2, acetyl-CoA and PamZ.

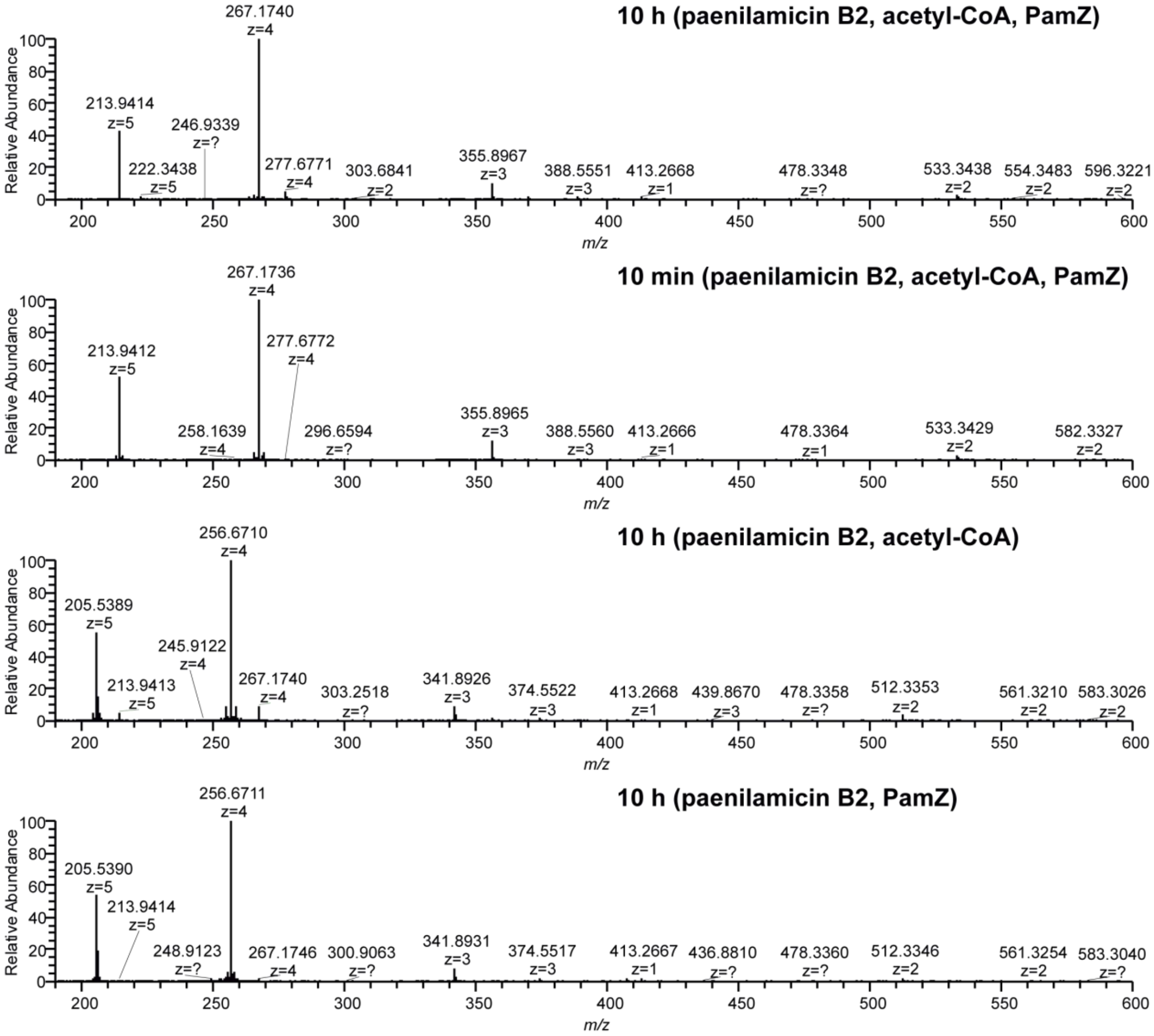

Supplementary Figure 7. MS[1] spectra of *in vitro* time-dependent activation assay including paenilamicin B2 (synthetic), acetyl-CoA and PamZ.

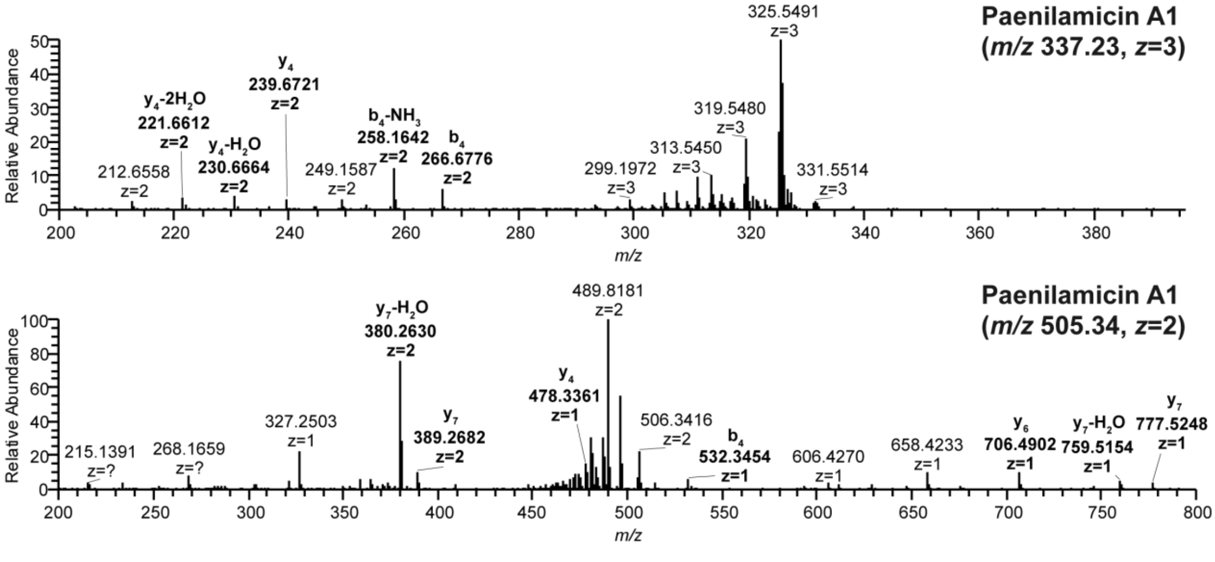

Supplementary Figure 8. MS[2] spectra of paenilamicin A1 isolated from *P. larvae* DSM 25430 (ERIC II).

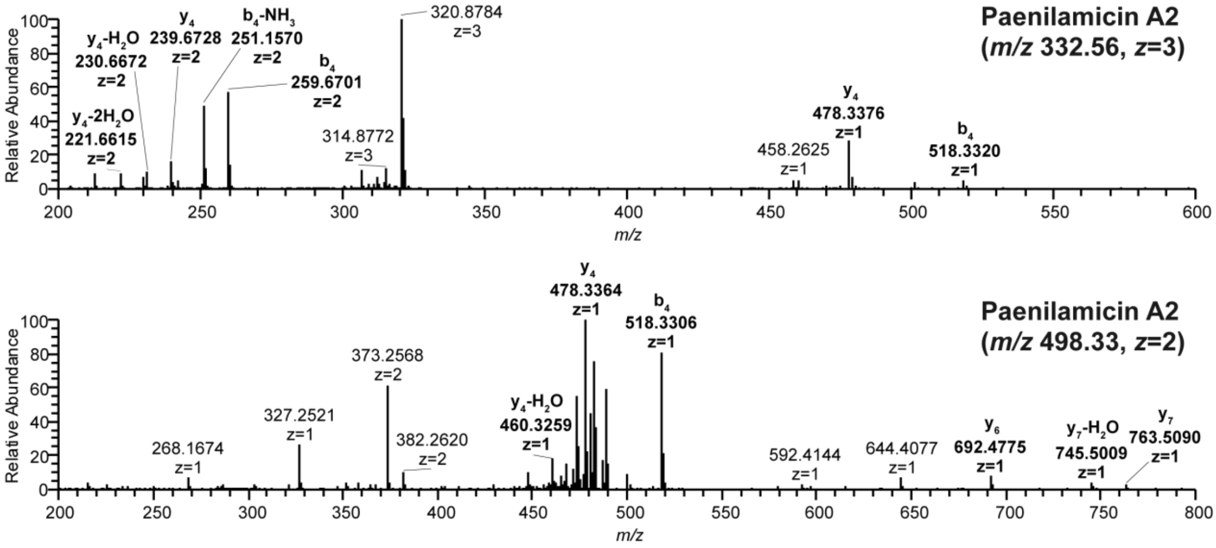

Supplementary Figure 9. MS^2 spectra of paenilamicin A2 isolated from *P. larvae* ATCC 9545 (ERIC I).

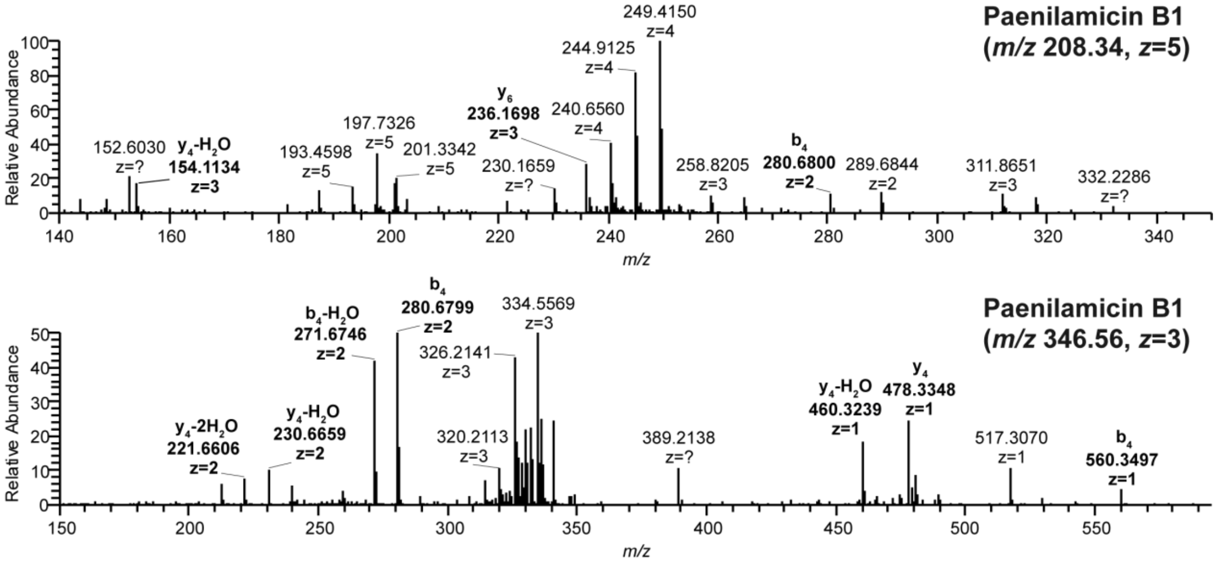

Supplementary Figure 10. MS^2 spectra of paenilamicin B1 isolated from *P. larvae* DSM 25430 (ERIC II).

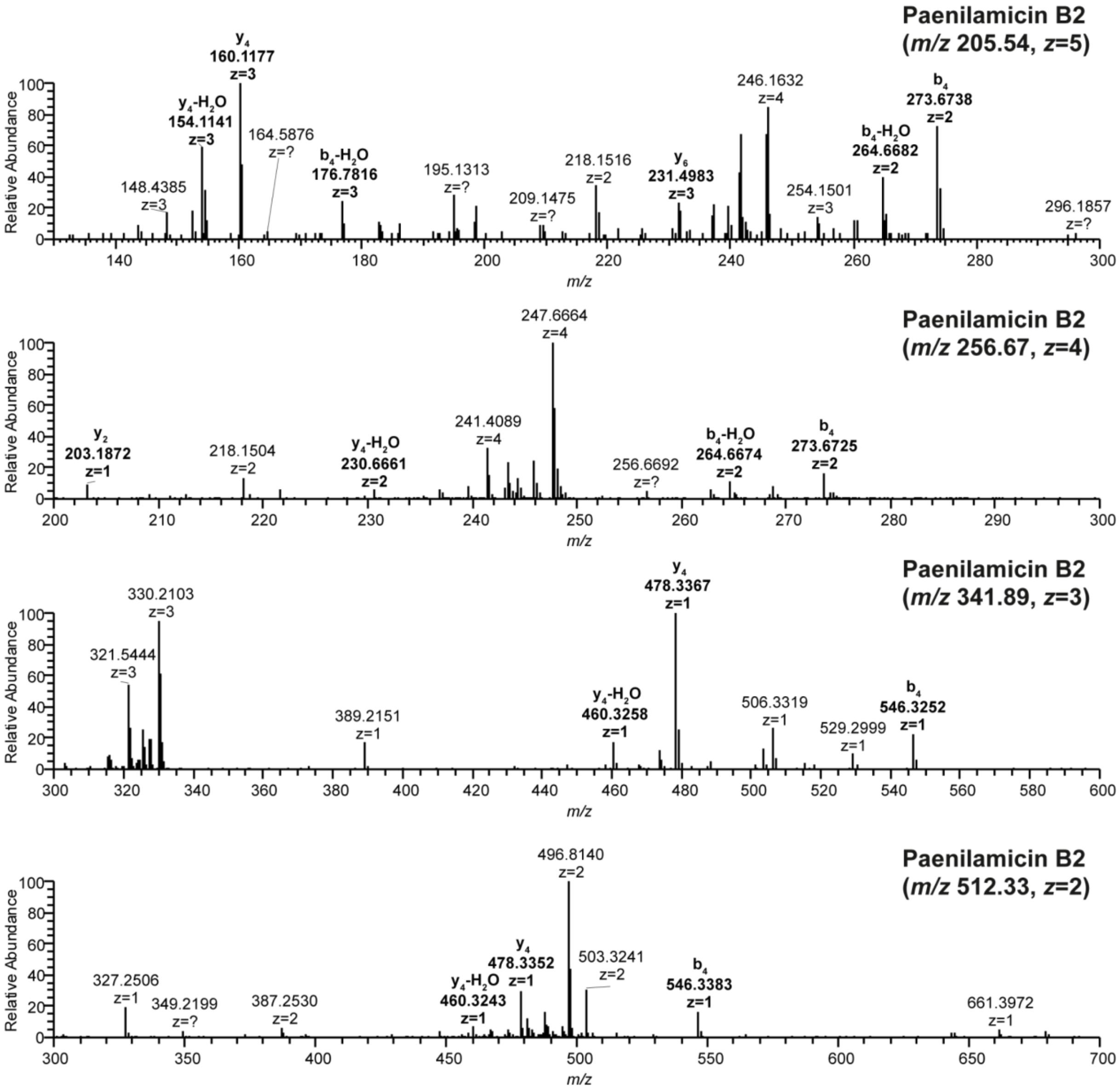

Supplementary Figure 11. MS^2 spectra of paenilamicin B2 isolated from *P. larvae* ATCC 9545 (ERIC I).

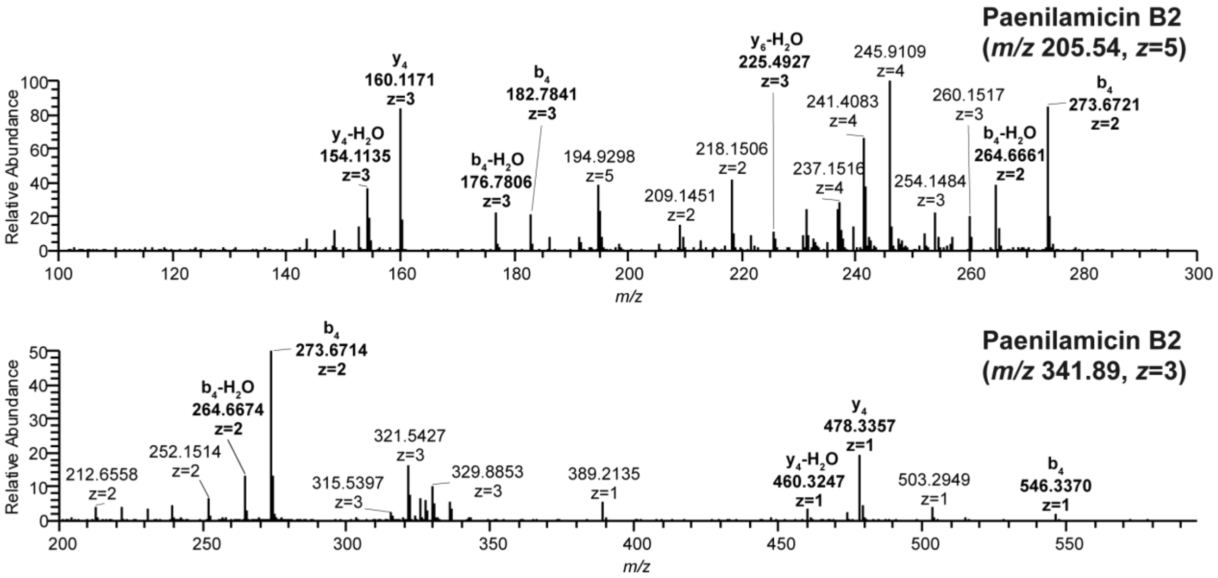

Supplementary Figure 12. MS^2 spectra of paenilamicin B2 (synthetic).

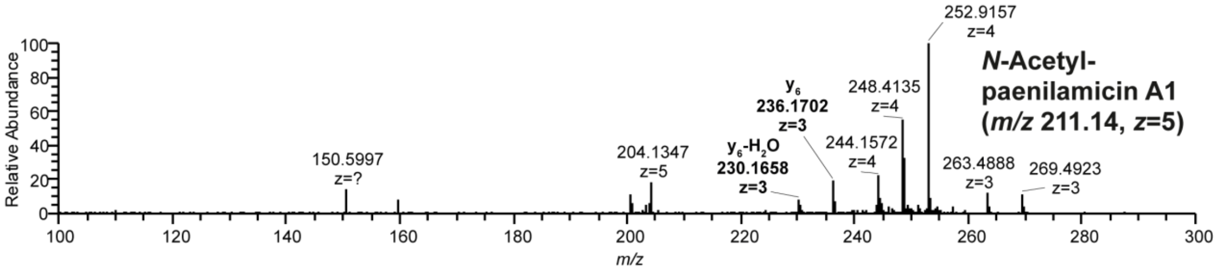

Supplementary Figure 13. MS^2 spectrum of *N*-acetylpaenilamicin A1 converted *in vitro* by PamZ.

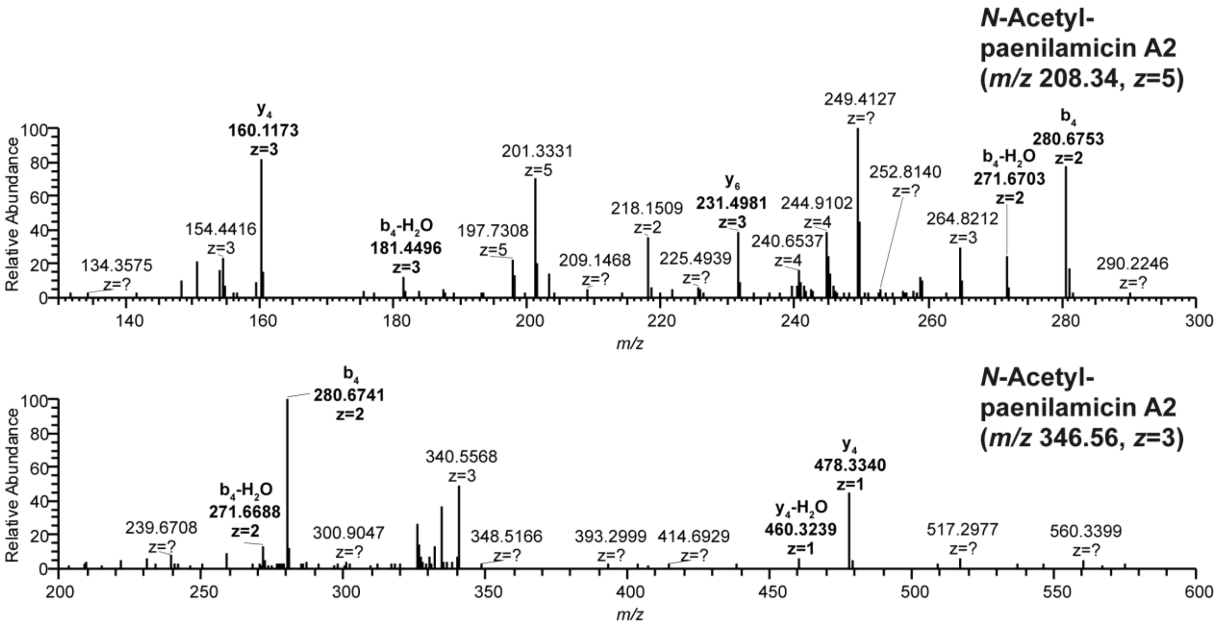

Supplementary Figure 14. MS^2 spectra of *N*-acetylpaenilamicin A2 converted *in vitro* by PamZ.

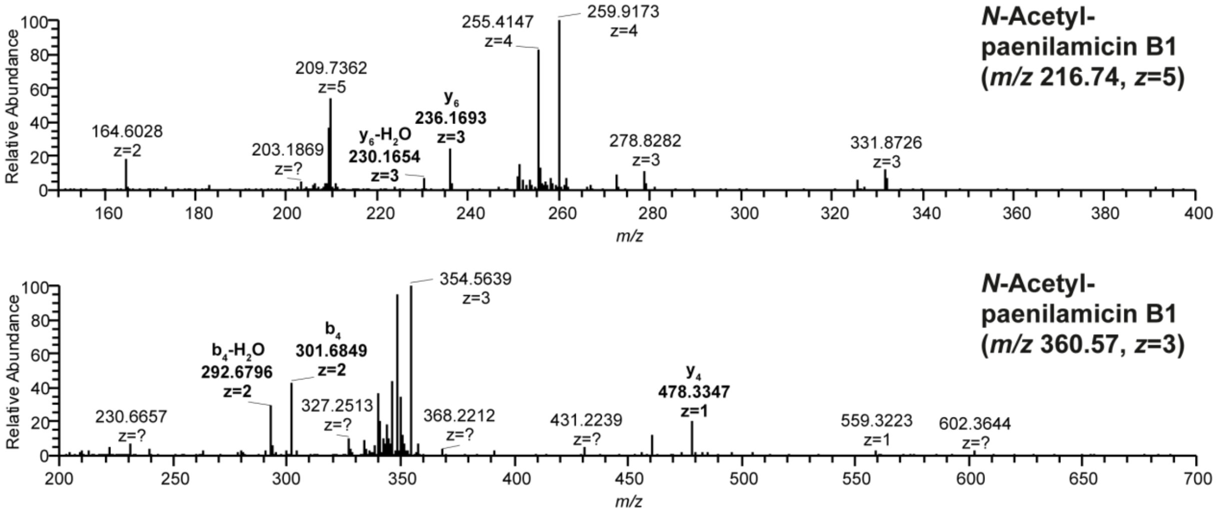

Supplementary Figure 15. MS^2 spectra of *N*-acetylpaenilamicin B1 converted *in vitro* by PamZ.

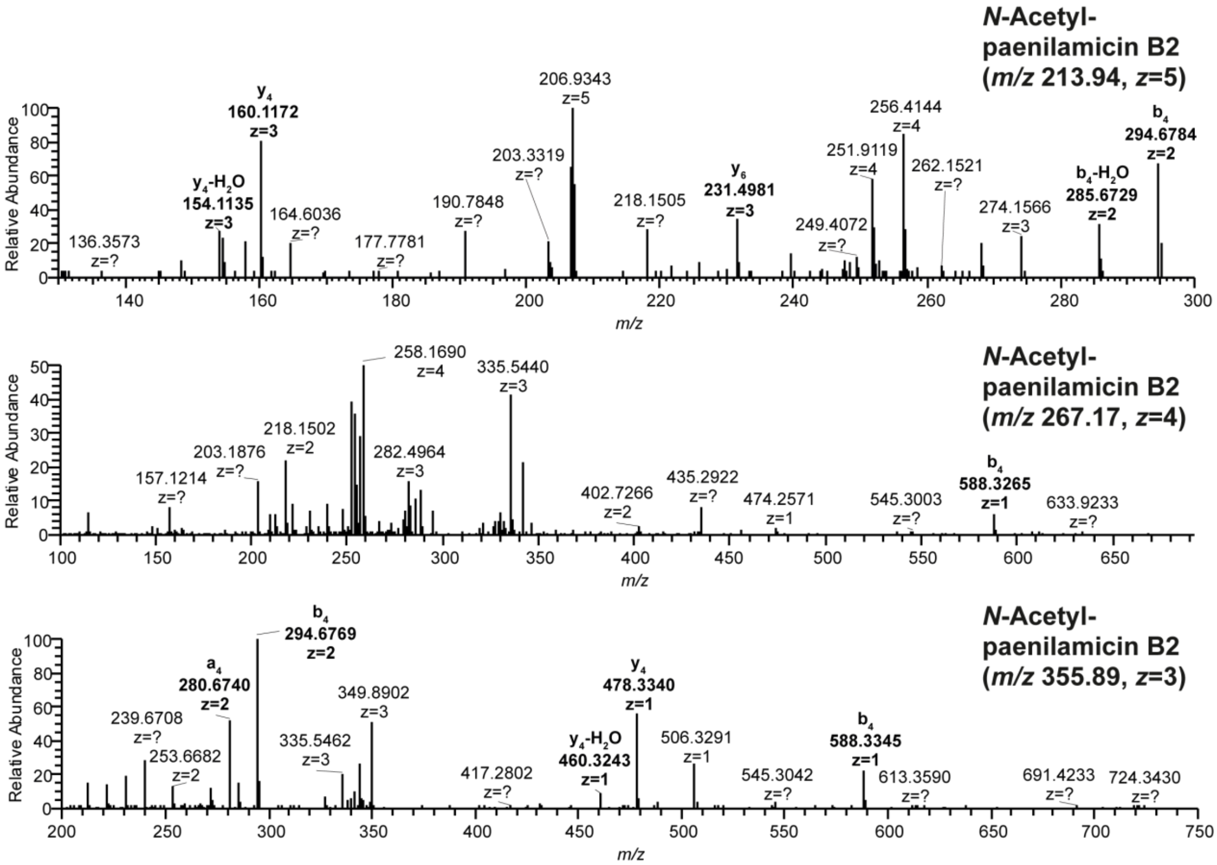

Supplementary Figure 16. MS^2 spectra of *N*-acetylpaenilamicin B2 converted *in vitro* by PamZ.

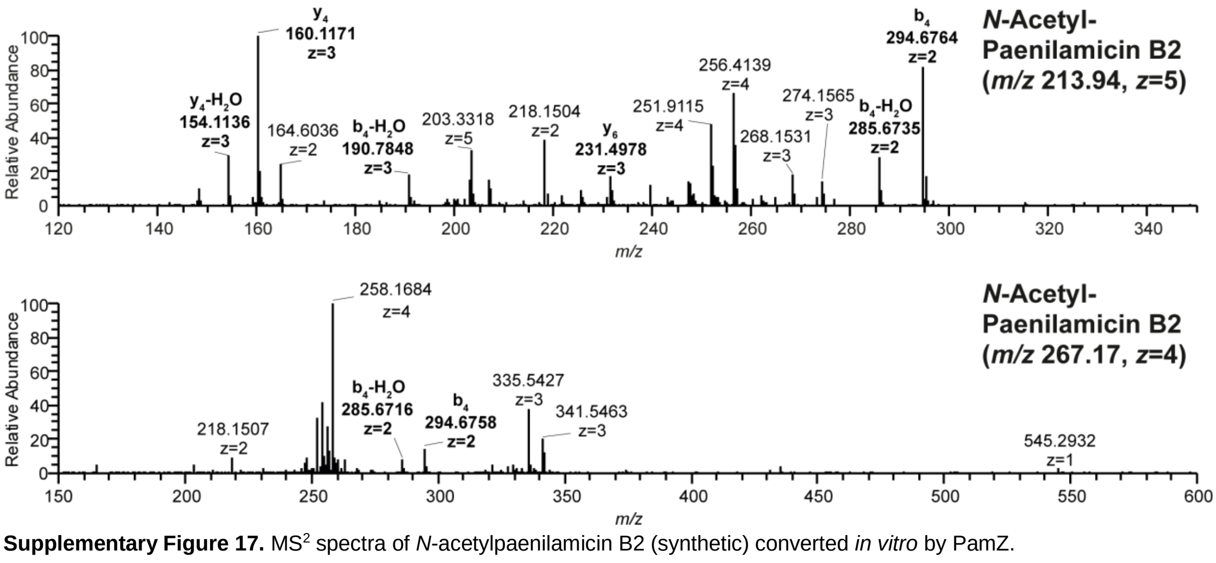

Supplementary Figure 17. MS^2 spectra of *N*-acetylpaenilamicin B2 (synthetic) converted *in vitro* by PamZ.

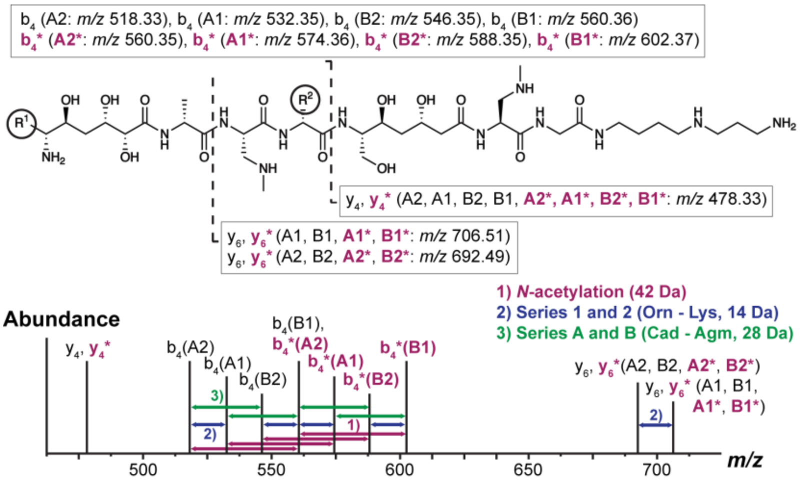

Derivative	Exact mass	b_4	y_4	y_6
A2	994.6499	518.33	478.33	692.48
A1	1008.6655	532.35	478.33	706.49
B2	1022.6560	546.35	478.33	231.50[2)]
B1	1036.6717	560.36	478.33	236.17[2)]
**A2***	**1036.6604**	**280.67[1)]**	**478.33**	**231.50[2)]**
**A1***	**1050.6761**	**574.33**	**478.33**	**236.17[2)]**
**B2***	**1064.6666**	**588.33**	**478.33**	**231.50[2)]**
**B1***	**1078.6822**	**602.36**	**478.33**	**236.17[2)]**

[1)] (z=2), [2)] (z=3)

Supplementary Figure 18. MS^2 fragmentation of different paenilamicin and *N*-acetylpaenilamicin variants to determine regioselective acetylation. Observed fragment ions b_4, y_4 and y_6 are highlighted in the chemical structure of paenilamicin. The different paenilamicin variants with residue R_1 (Glm, Aga) and R_2 (Orn, Lys) are shown as circles. Fragment ions of *N*-acetylpaenilamicin variants are indicated bold (magenta) and with an asterisk. The schematic MS^2 spectrum shows the observed fragment ions of each single paenilamicin and *N*-acetylpaenilamicin variant. Arrows show distinct mass shifts of acetylation (magenta) and the different paenilamicin series (blue and green). Observed fragment ions are listed as mass-to-charge ratios with z=1.

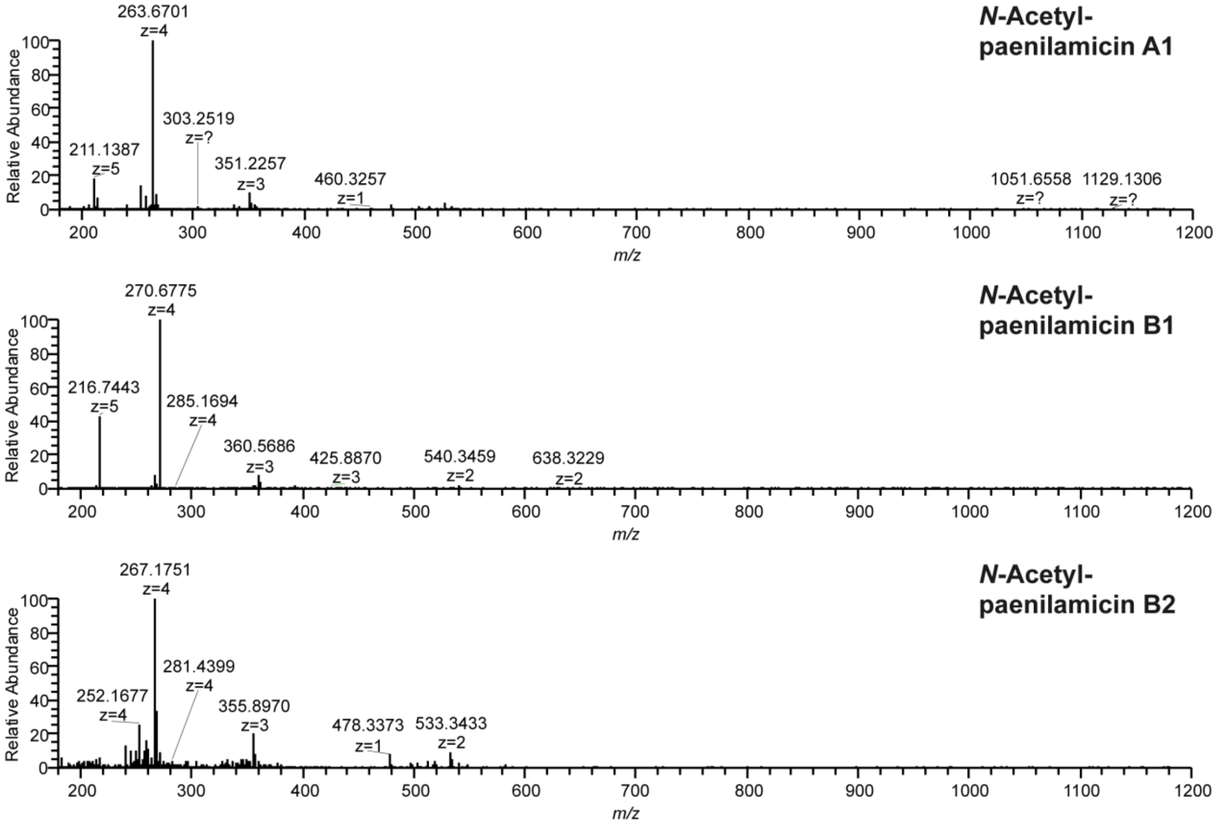

Supplementary Figure 19. MS^1 spectra of *N*-acetylpaenilamicin A1, B1 and B2 isolated from *P. larvae*.

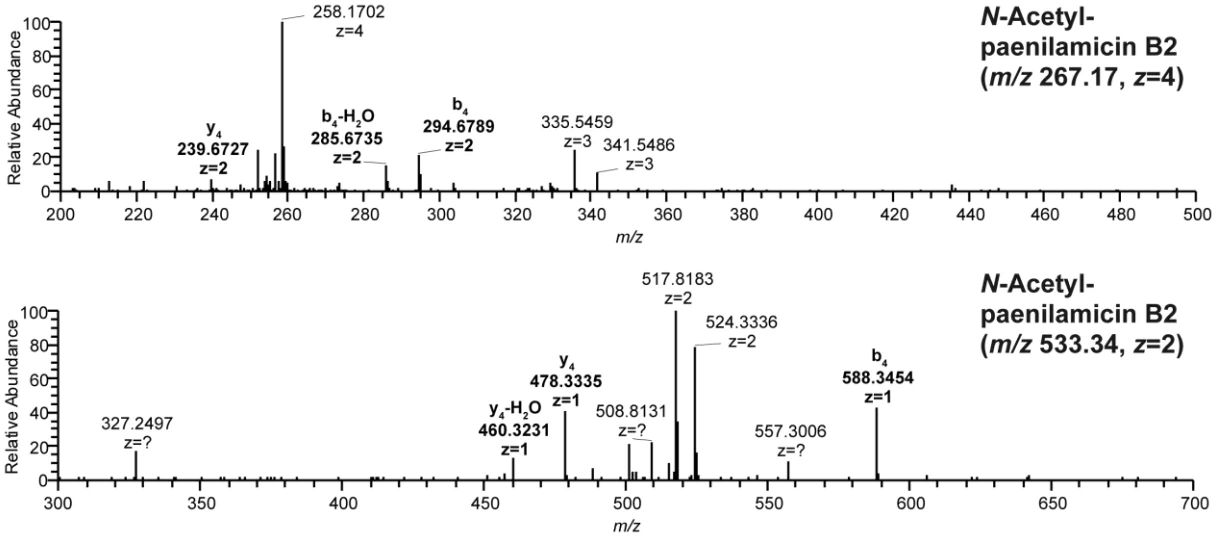

Supplementary Figure 20. MS^2 spectra of *N*-acetylpaenilamicin B2 isolated from *P. larvae* DSM 25430 (ERIC II).

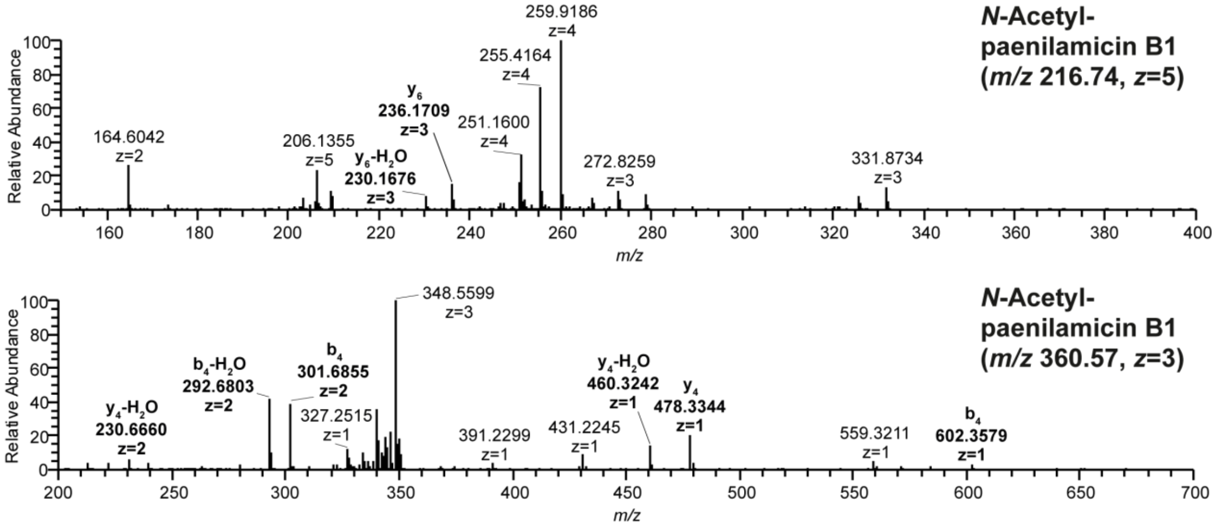

Supplementary Figure 21. MS^2 spectra of *N*-acetylpaenilamicin B1 isolated from *P. larvae* DSM 25430 (ERIC II).

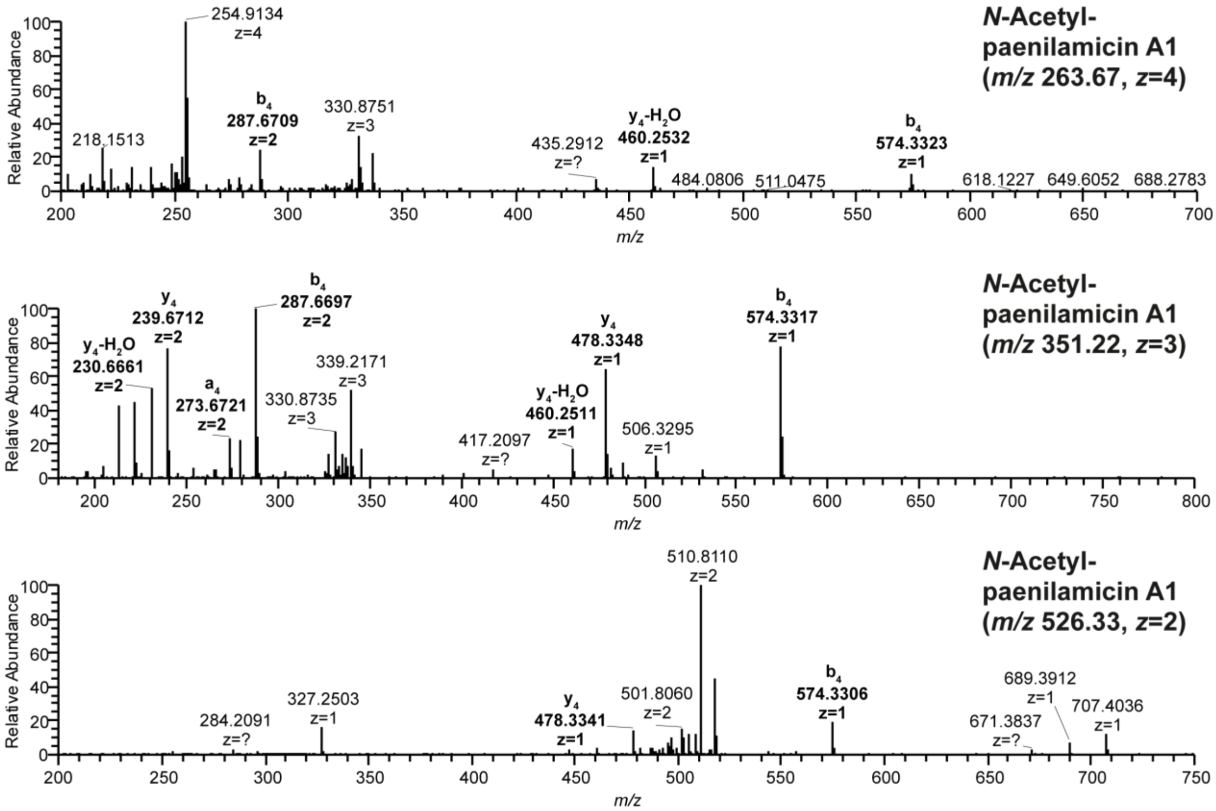

Supplementary Figure 22. MS^2 spectra of *N*-acetylpaenilamicin A1 isolated from *P. larvae* ATCC 9545 (ERIC I).

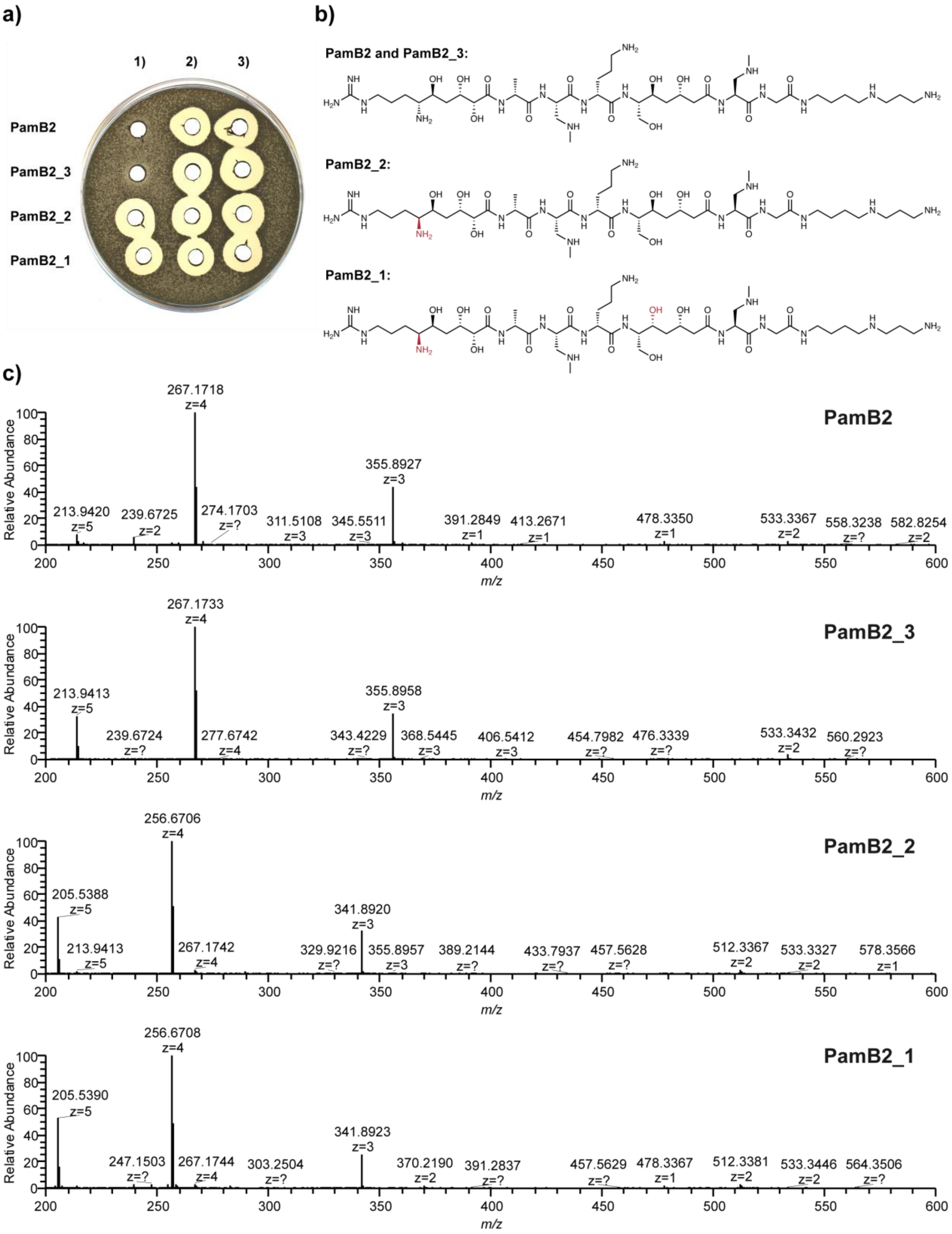

Supplementary Figure 23. Stereoselective *N*-acetylation of PamZ. a) Natural paenilamicin B2 (**PamB2**), synthetic paenilamicin B2 (**PamB2_3**) and its diastereomers (**PamB2_2**, **PamB2_1**) were incubated with acetyl-CoA and PamZ (1), only acetyl-CoA (2), only PamZ (3) *in vitro* and tested by agar diffusion assay against *B. megaterium* as indicator strain. **b)** Chemical structure of paenilamicin B2 and its diastereomers. **c)** Reaction monitored and verified by HPLC-ESI MS.

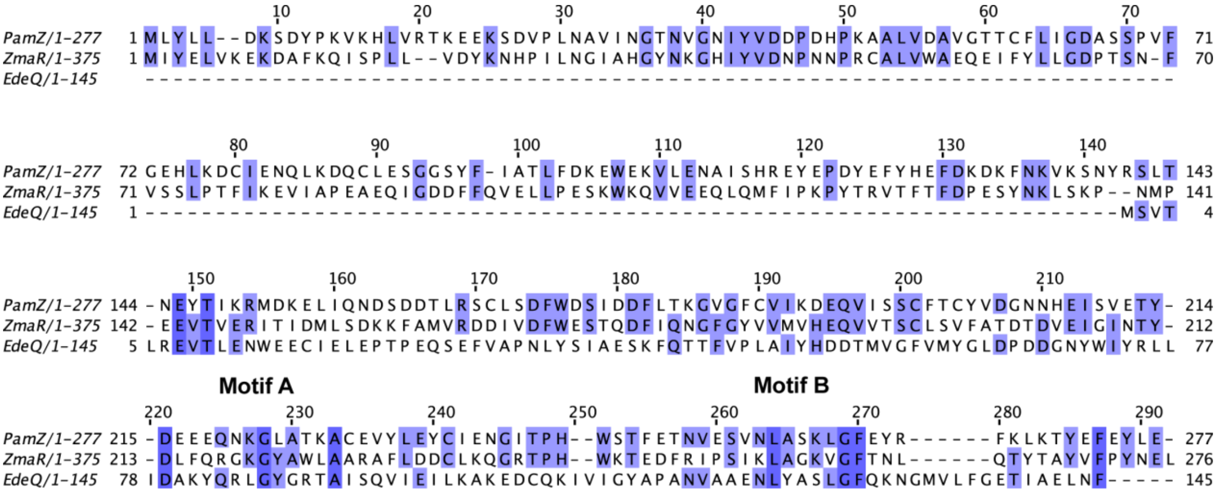

Supplementary Figure 24. Multiple sequence alignment of Gcn5-related *N*-acetyltransferases of PamZ (paenilamicin), ZmaR (zwittermicin) and EdeQ (edeine). Blue shaded residues are identical. The pyrophosphate group of coenzyme A interacts with the highly conserved motif A and the adenine ring with the conserved motif B.

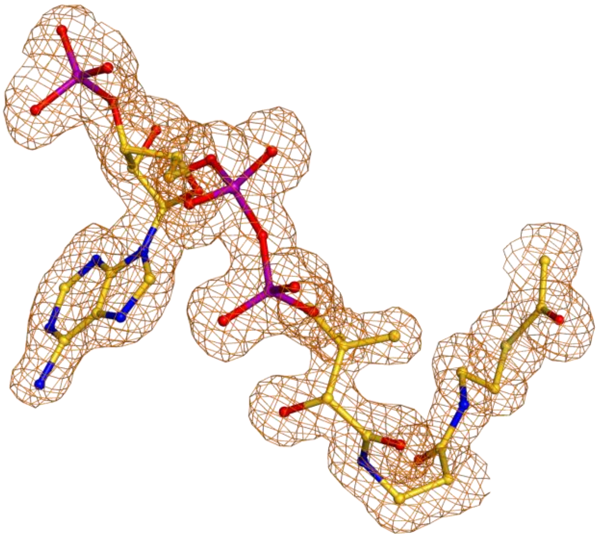

Supplementary Figure 25. Polder electron density map[1] of acetyl-CoA shown as mesh at a σ-level of 3.0. Acetyl-CoA is presented as ball-stick-model with carbon atoms colored in magenta, oxygen in red, phosphorous in orange and nitrogen in light blue. Hydrogen atoms are not shown.

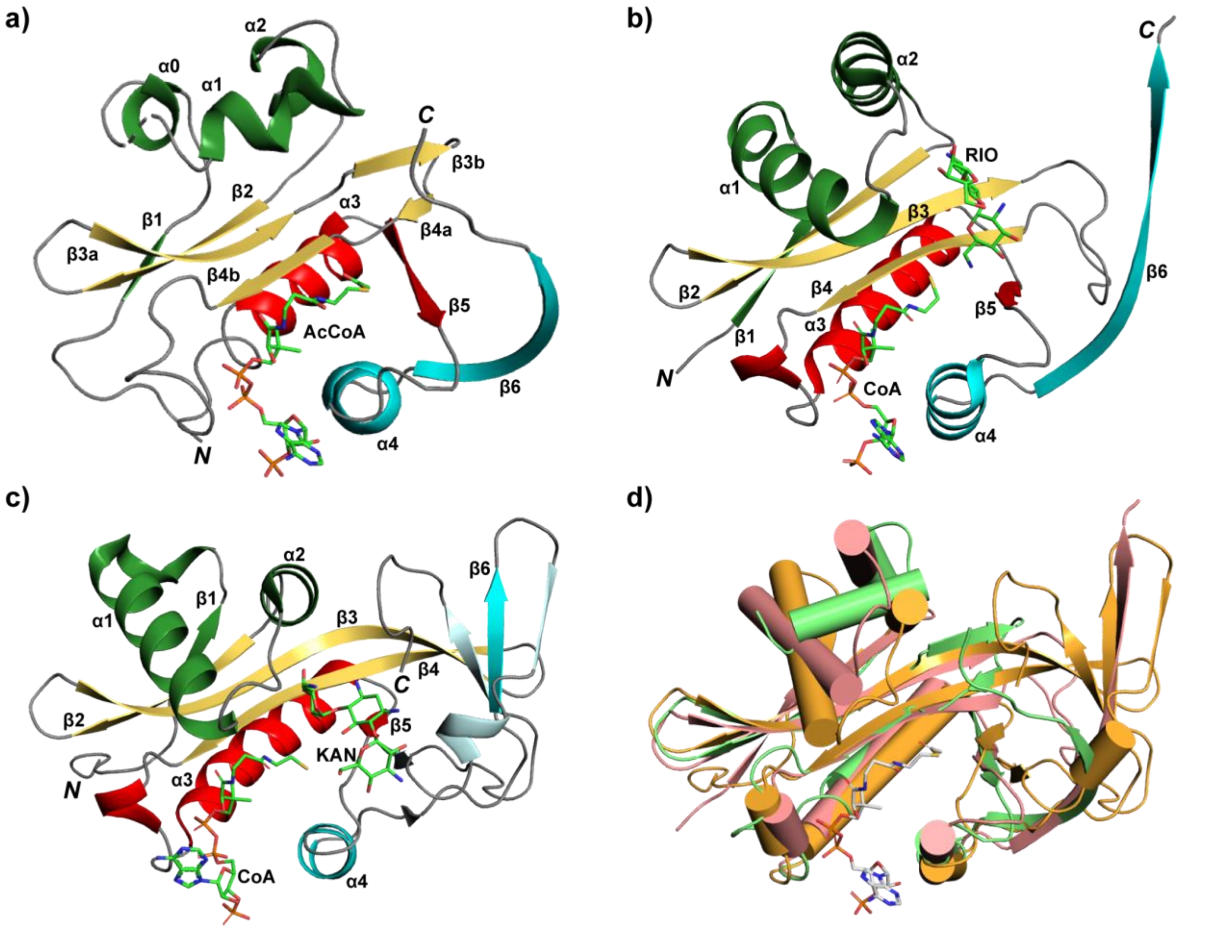

Supplementary Figure 26. Structural comparison of PamZ and AACs. a) C-terminal domain of PamZ including acetyl-CoA, **b)** AAC(6') from *Salmonella enterica* including CoA and ribostamycin (PDB ID: 1s3z), **c)** AAC(2') from *Mycobacterium tuberculosis* including CoA and kanamycin A (PDB ID: 1m4i), **d)** pairwise structural alignment of C-terminal domain of PamZ (green), AAC(6') (pink) and AAC(2') (orange). The CTD of PamZ superimposes with the AAC(6') with an RMSD of 1.9 Å for 104 pairs of Cα-atoms. AAC(2') superimposes with CTD of PamZ with an RMSD of 4.0 Å for 75 pairs of Cα-atoms.

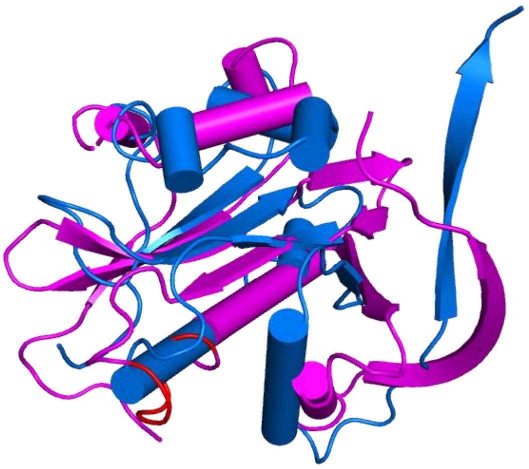

Supplementary Figure 27. Pairwise structural alignment between NTD and CTD of PamZ. The NTD is highlighted in magenta and CTD in blue. The P-loop only present in CTD is highlighted in red.

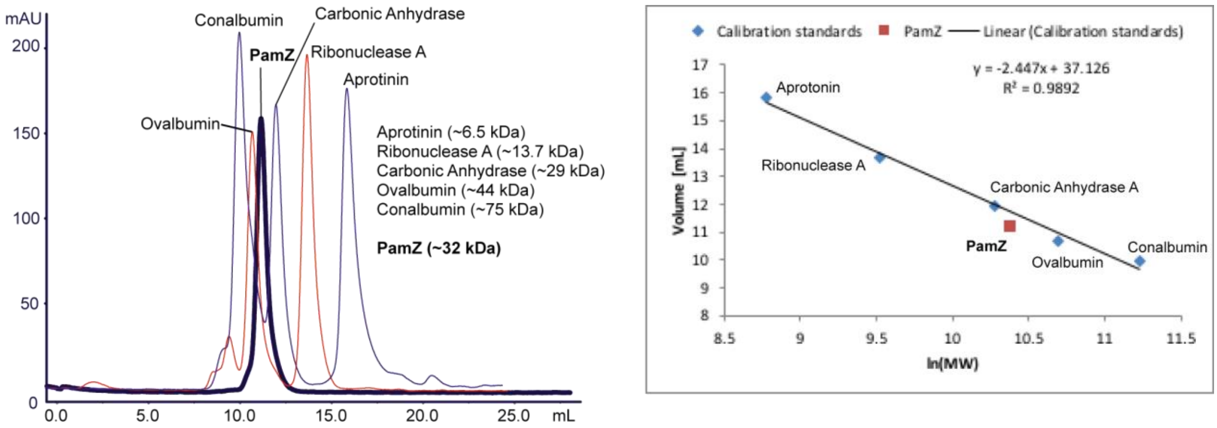

Supplementary Figure 28. Size exclusion chromatogram and calibration curve of PamZ including calibration standards detected at 280 nm. Chromatogram indicates PamZ as monomeric unit.

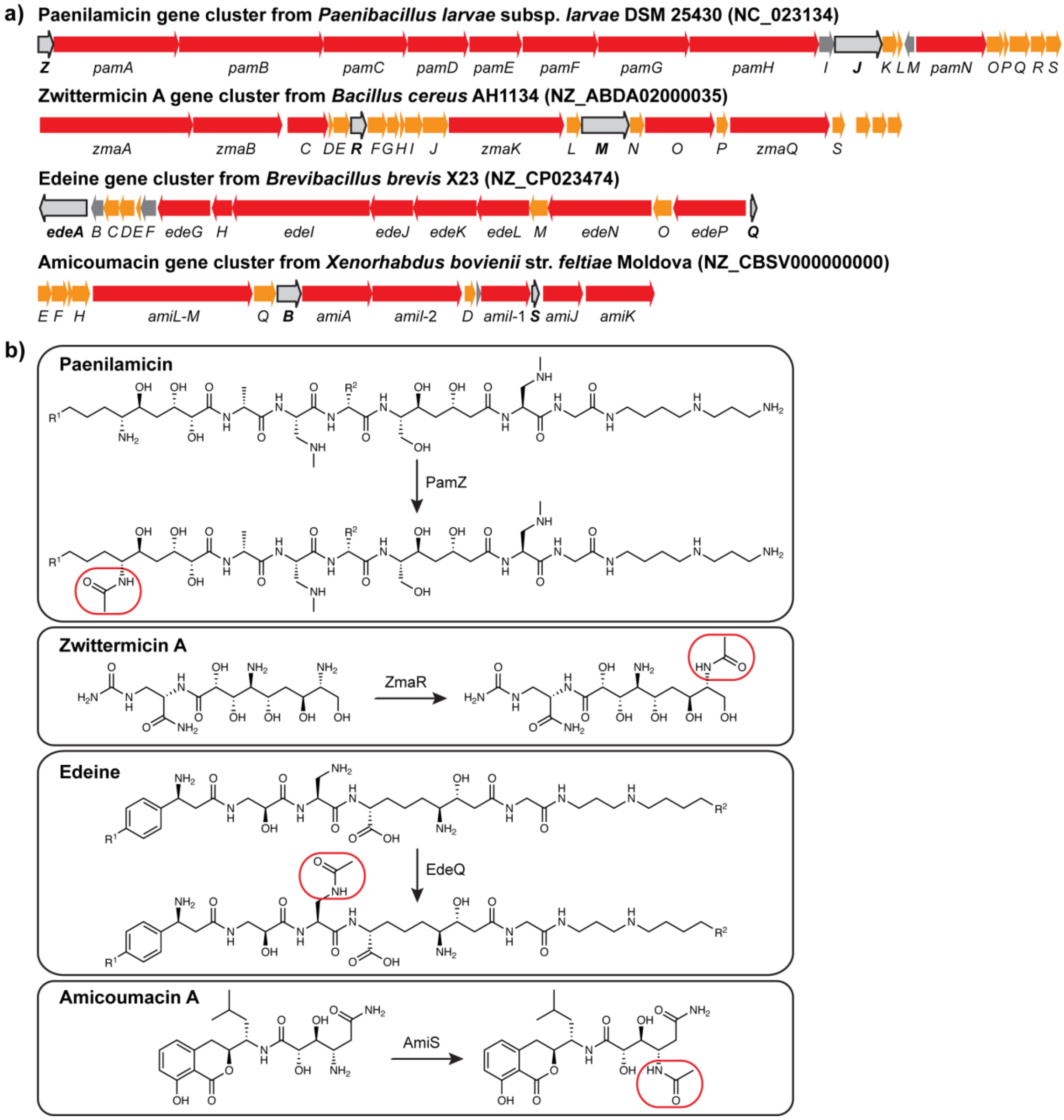

Supplementary Figure 29. Biosynthetic gene cluster and *N*-acetylation of paenilamicin, zwittermicin A, edeine and amicoumacin A. a) Different functions of the genes are grouped by color: core biosynthetic gene (red), additional biosynthetic

gene (orange), other gene (dark grey), resistance gene (light grey and framed in black). **b)** *N*-acetylation reaction of the cationic peptides. Position of *N*-acetylation framed in red.

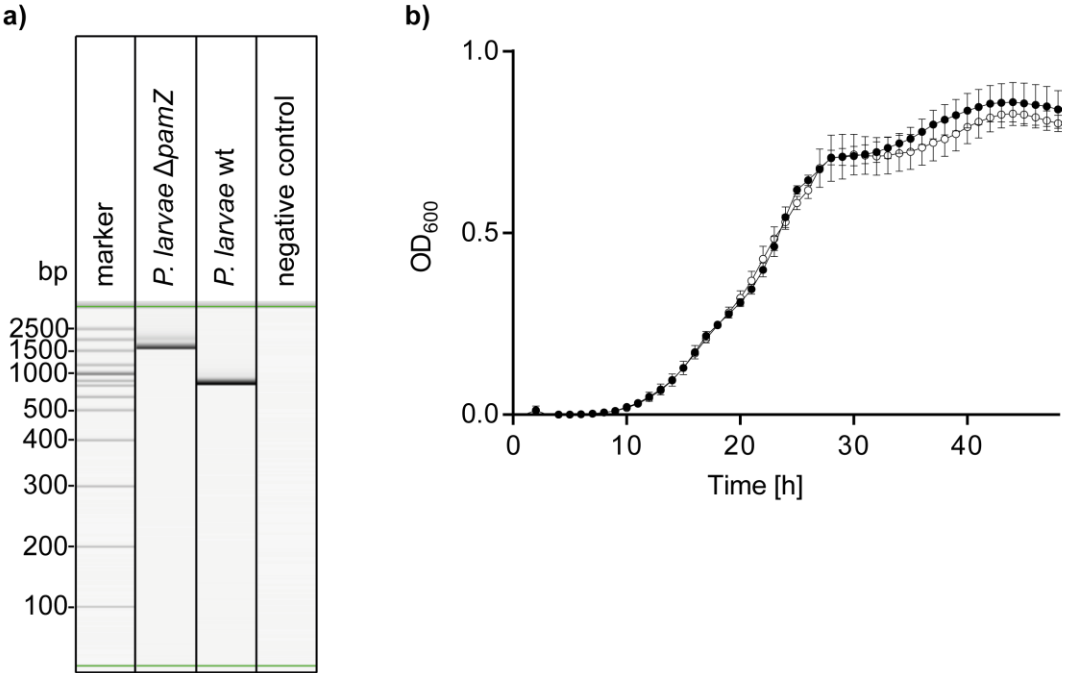

Supplementary Figure 30. Determination of successful intron insertion into the *pamZ* gene of *P. larvae* DSM 25430. **a)** PCR analysis with subsequent capillary gel electrophoresis (QIAxcel Advanced System with QIAxcel ScreenGel software v1.5.0.16, Qiagen, Hilden, Germany) revealed an about 900 bp larger fragment caused by intron insertion in the knockout mutant Δ*pamZ* (1612 bp) compared with the wild type strain (712 bp). **b)** Growth curves of *P. larvae* WT (black circles) and the *P. larvae* Δ*pamZ* deletion mutant (white circles) in MYPGP liquid broth under anaerobic conditions (two-way-ANOVA, p=0.6486). This experiment was repeated three times with three biological replicates with three technical replicates each, n=3 biologically independent samples. Data are presented as mean values +/- SEM. Source data are provided as a Source Data file.

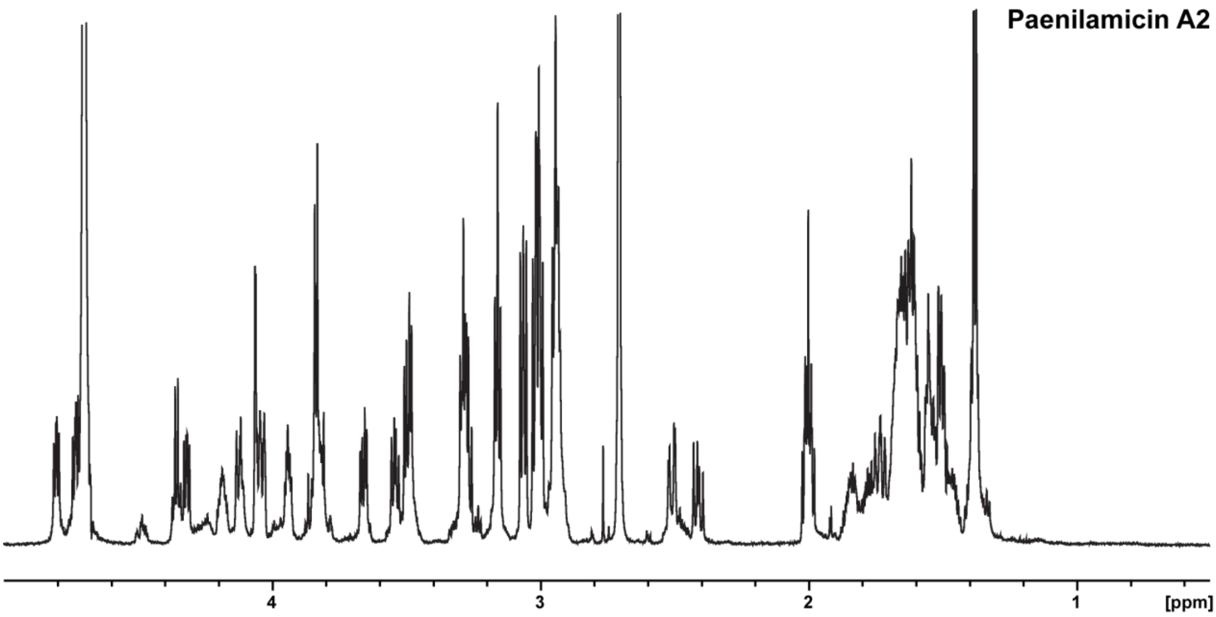

Supplementary Figure 31. ^{1}H-NMR spectrum of paenilamicin A2 isolated from *P. larvae* ATCC 9545 and recorded in D_2O at 298 K displays aliphatic region.

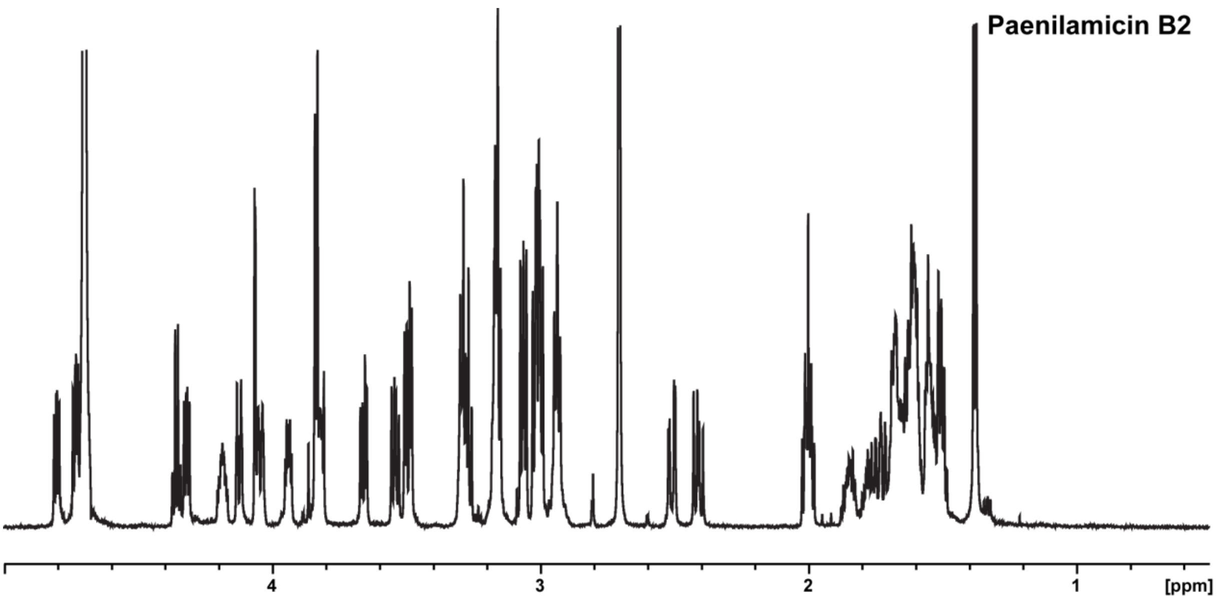

Supplementary Figure 32. ^{1}H-NMR spectrum of paenilamicin B2 isolated from *P. larvae* ATCC 9545 and recorded in D_2O at 298 K displays aliphatic region.

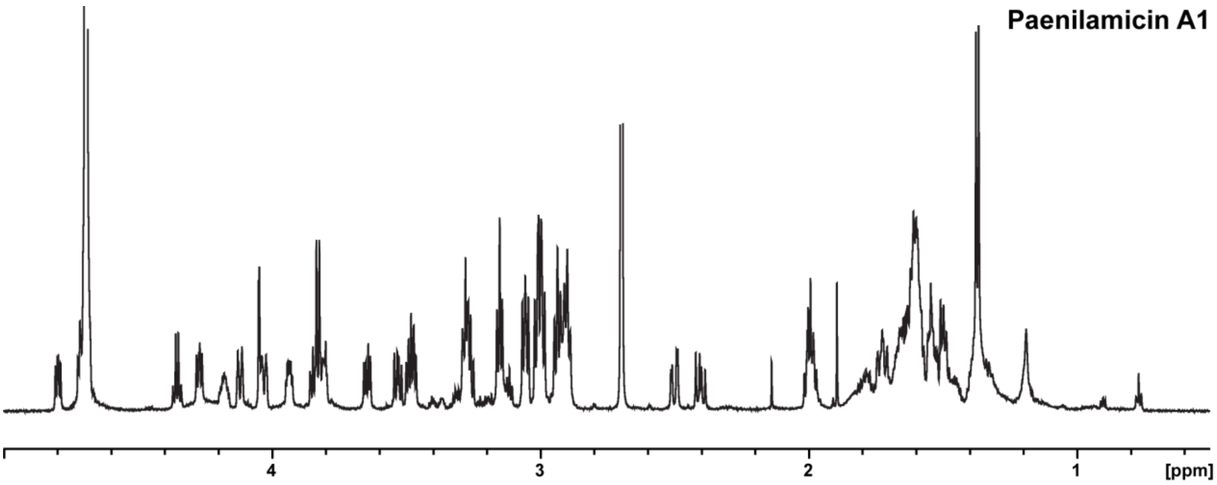

Supplementary Figure 33. ^{1}H-NMR spectrum of paenilamicin A1 isolated from *P. larvae* DSM 25430 and recorded in D_2O at 298 K displays aliphatic region.

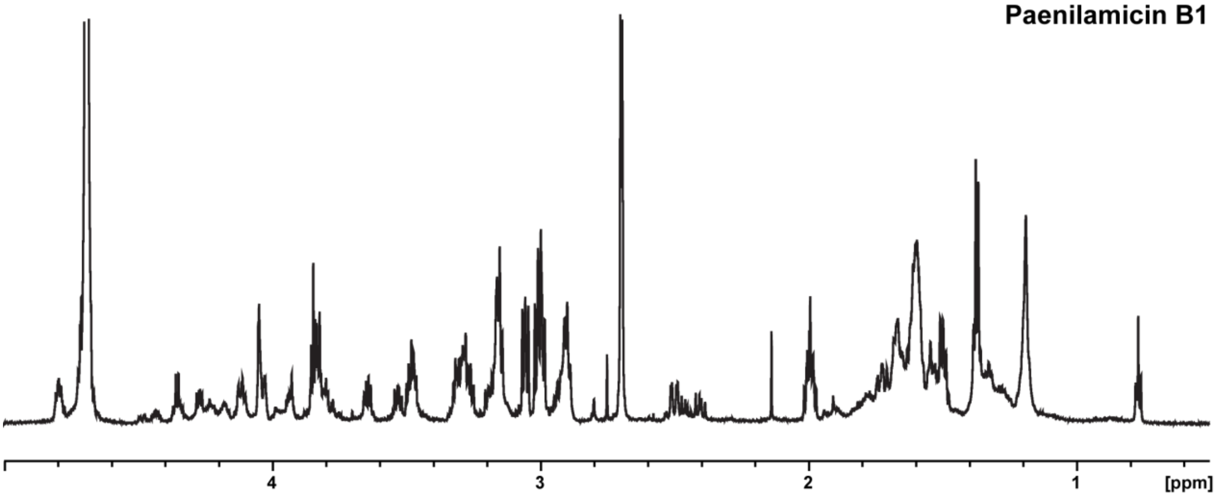

Supplementary Figure 34. ^{1}H-NMR spectrum of paenilamicin B1 isolated from *P. larvae* DSM 25430 and recorded in D_2O at 298 K displays aliphatic region.

Supplementary Tables

Supplementary Table 1. Fragment ions of paenilamicin, *N*-acetylpaenilamicin and N^2-acetylpaenilamicin variants.

Supplementary Table 2. Comparison of ^{1}H and ^{13}C chemical shifts of paenilamicin B2 and *N*-acetylpaenilamicin B2 in D_2O and 0.1% acetic acid-d_4 at 298 K.

Supplementary Table 3. Crystallographic data collection and model refinement statistics for binary complex PamZ including acetyl-CoA.

Supplementary Table 4. Primers used for knockout generation and screening for knockout mutants.

Supplementary Table 5. Oligonucleotides used in this study.

Supplementary Table 1. Fragment ions of paenilamicin, *N*-acetylpaenilamicin and N^2-acetylpaenilamicin variants.

Compound	Observed *m/z*	Calculated *m/z*	Fragment ions
Paenilamicin A1 (isolated)	*m/z* 337.23 (*z*=3)<br>*m/z* 505.34 (*z*=2)	*m/z* 337.23 (*z*=3)<br>*m/z* 505.34 (*z*=2)	b_4, y_4<br>b_4, y_4, y_6, y_7
Paenilamicin A2 (isolated)	*m/z* 332.56 (*z*=3)<br>*m/z* 498.33 (*z*=2)	*m/z* 332.56 (*z*=3)<br>*m/z* 498.33 (*z*=2)	b_4, y_4<br>b_4, y_4, y_6, y_7
Paenilamicin B1 (isolated)	*m/z* 208.34 (*z*=5)<br>*m/z* 346.56 (*z*=3)	*m/z* 208.34 (*z*=5)<br>*m/z* 346.56 (*z*=3)	b_4, y_4, y_6<br>b_4, y_4
Paenilamicin B2 (isolated)	*m/z* 205.54 (*z*=5)<br>*m/z* 256.67 (*z*=4)<br>*m/z* 341.89 (*z*=3)<br>*m/z* 512.33 (*z*=2)	*m/z* 205.54 (*z*=5)<br>*m/z* 256.67 (*z*=4)<br>*m/z* 341.89 (*z*=3)<br>*m/z* 512.34 (*z*=2)	b_4, y_4, y_6<br>b_4, y_2, y_4<br>b_4, y_4<br>b_4, y_4
Paenilamicin B2 (synthesized)	*m/z* 205.54 (*z*=5)<br>*m/z* 341.89 (*z*=3)	*m/z* 205.54 (*z*=5)<br>*m/z* 341.89 (*z*=3)	b_4, y_4, y_6<br>b_4, y_4
N-acetylpaenilamicin A1 (isolated, *in vitro*)	*m/z* 211.14 (*z*=5)	*m/z* 211.14 (*z*=5)	y_6
N^2-acetylpaenilamicin A1 (isolated, *in vitro*)	*m/z* 365.24 (*z*=3)	*m/z* 365.24 (*z*=3)	b_4, y_4
N-acetylpaenilamicin A2 (isolated, *in vitro*)	*m/z* 208.34 (*z*=5)<br>*m/z* 346.56 (*z*=3)	*m/z* 208.34 (*z*=5)<br>*m/z* 346.56 (*z*=3)	b_4, y_4, y_6<br>b_4, y_4
N^2-acetylpaenilamicin A2 (isolated, *in vitro*)	*m/z* 360.57 (*z*=3)	*m/z* 360.56 (*z*=3)	b_4, y_4
N-acetylpaenilamicin B1 (isolated, *in vitro*)	*m/z* 216.74 (*z*=5)<br>*m/z* 360.57 (*z*=3)	*m/z* 216.74 (*z*=5)<br>*m/z* 360.57 (*z*=3)	y_6<br>b_4, y_4
N-acetylpaenilamicin B2 (isolated, *in vitro*)	*m/z* 213.94 (*z*=5)<br>*m/z* 267.17 (*z*=4)<br>*m/z* 355.89 (*z*=3)	*m/z* 213.94 (*z*=5)<br>*m/z* 267.17 (*z*=4)<br>*m/z* 355.90 (*z*=3)	b_4, y_4, y_6<br>b_4<br>a_4, b_4, y_4
N-acetylpaenilamicin B2 (synthesized, *in vitro*)	*m/z* 213.94 (*z*=5)<br>*m/z* 267.17 (*z*=4)	*m/z* 213.94 (*z*=5)<br>*m/z* 267.17 (*z*=4)	b_4, y_4, y_6<br>b_4
N-acetylpaenilamicin A1 (isolated)	*m/z* 263.67 (*z*=4)<br>*m/z* 351.22 (*z*=3)<br>*m/z* 526.33 (*z*=2)	*m/z* 263.68 (*z*=4)<br>*m/z* 351.23 (*z*=3)<br>*m/z* 526.35 (*z*=2)	b_4, y_4<br>a_4, b_4, y_4<br>b_4, y_4
N-acetylpaenilamicin B1 (isolated)	*m/z* 216.74 (*z*=5)<br>*m/z* 360.57 (*z*=3)	*m/z* 216.74 (*z*=5)<br>*m/z* 360.57 (*z*=3)	y_6<br>b_4, y_4
N-acetylpaenilamicin B2 (isolated)	*m/z* 267.17 (*z*=4)<br>*m/z* 533.34 (*z*=2)	*m/z* 267.17 (*z*=4)<br>*m/z* 533.34 (*z*=2)	b_4, y_4<br>b_4, y_4

Supplementary Table 2. Comparison of ^{1}H and ^{13}C chemical shifts of paenilamicin B2 and *N*-acetylpaenilamicin B2 in D_2O and 0.1% acetic acid-d_4 at 298 K. Chemical shifts and chemical shift perturbations (CSPs) are given in ppm. The color code refers to that used in Fig. 3b of the main manuscript.

		Paenilamicin B2		*N*-acetylpaenilamicin B2		
**Residue**	**Pos.**	**δ ^{1}H**	**δ ^{13}C**	**δ ^{1}H**	**δ ^{13}C**	**CSP**
**Aga**	**2**	4.15	77.26	4.13	77.40	0.03
	**3**	4.21	71.00	4.20	71.35	0.03
	**4'**	1.82	37.78	1.82	38.66	0.05
	**4''**	1.63		1.52		0.12
	**5**	4.13	69.78	3.77	72.41	0.39
	**6**	3.38	58.64	3.82	56.76	0.45
	**7'**	1.74	26.76	1.74	28.82	0.12
	**7''**	1.67		1.44		0.26
	**8'**	1.75	27.22	1.66	27.34	0.09
	**8''**	1.67		1.56		0.10
	**9**	3.26	43.38	3.20	43.49	0.06
**Ac**	**2**	n.d.[a]	n.d.[a]	2.04	24.71	n.d.[a]
**Ala**	α	4.44	52.38	n.d.[a]	n.d.[a]	n.d.[a]
	β	1.47	19.06	1.46	18.90	0.01
**mDap1**	α	4.82	52.73	n.d.[a]	n.d.[a]	n.d.[a]
	β'	3.58	51.63	3.58	51.63	0.00
	β''	3.38		3.38		0.00
	δ	2.79	36.20	2.79	36.18	0.00
**Orn**	α	4.41	56.52	n.d.[a]	n.d.[a]	n.d.[a]
	β'	1.94	30.86	1.94	30.85	0.00
	β''	1.87		1.87		0.00
	γ	1.74	26.04	1.73	26.02	0.01
	δ	3.03	41.58	3.02	41.59	0.00
**Gla**	**2'**	2.59	46.36	2.60	46.35	0.00
	**2''**	2.51		2.50		0.01
	**3**	4.27	68.03	4.27	68.02	0.00
	**4**	1.64	43.05	1.64	43.08	0.00
	**5**	4.03	68.97	4.03	68.95	0.01
	**6**	3.91	58.77	3.90	58.77	0.01
	**7'**	3.75	63.76	3.74	63.79	0.01
	**7''**	3.63		3.63		0.00
**mDap2**	α	4.89	52.43	n.d.[a]	n.d.[a]	n.d.[a]
	β'	3.58	51.81	3.58	51.81	0.00
	β''	3.36		3.36		0.00
	δ	2.79	36.20	2.79	36.18	0.00
**Gly**	α	3.92	45.24	3.92	45.22	0.00
**Spd**	**2**	3.11	39.27	3.10	39.27	0.00
	**3**	2.09	26.45	2.09	26.48	0.01
	**4**	3.15	47.18	3.15	47.19	0.00
	**6**	3.09	50.07	3.09	50.07	0.00
	**7**	1.70	25.69	1.71	25.69	0.00
	**8**	1.59	28.20	1.59	28.24	0.00
	**9**	3.25	41.36	3.25	41.37	0.00

[a] not determined.

Supplementary Table 3. Crystallographic data collection and model refinement statistics for binary complex PamZ including acetyl-CoA.

**Data Collection**	
PDB ID	7B3A
Wavelength [Å]	0.91841
Temperature [K]	100
Space group	$P2_1$
**Unit cell parameters**	
a, b, c [Å]	36.4, 70.1, 54.6
α, β, γ [°]	90.0, 107.0, 90.0
Resolution range [Å][a]	50.00-1.34 (1.42-1.34)
**Reflections[a]**	
Unique	58,898 (9,129)
Completeness [%]	98.2 (94.5)
Multiplicits	3.8 (3.7)
**Data quality[a]**	
Intensity [I/σ(I)]	11.20 (1.00)
R_{meas} [%][b]	6.8 (129.2)
$CC_{1/2}$[c]	99.9 (37.9)
Wilson B value [$Å^2$]	23.1
**Refinement**	
Resolution range [Å][a]	50.00-1.34 (1.37-1.34)
**Reflections[a]**	
Number	58,882 (3,578)
Test set (3.6%)	2,099 (127)
R_{work}[a]	0.147 (0.305)
R_{free}[a]	0.181 (0.358)
**Contents of the asymmetric unit**	
Protein, molecules, residues, atoms	1, 1, 431, 3774
Acetyl-CoA, acetate molecules, chloride	1, 1, 1
Water, molecules	270
**Mean temperature factors [$Å^2$][b]**	
All Atoms	24.5
Macromolecules	23.8
Ligands	28.0
Water oxygens	32.5
**RMSD[d] from target geometry**	
Bond lengths [Å]	0.016
Bond angles [°]	1.500
**Validation statistics[c]**	
Ramachandran plot[e]	
Residues in allowed regions [%]	1.8
Residues in favored regions [%]	98.2
Rotamer outliers [%][e]	1.0
MOLPROBITY clashscore[f,g]	3.0
MOLPROBITY overall[f]	1.1

[a] data for the highest resolution shell in parenthesis.

[b] $R_{meas}(I) = \sum_h [N/(N-1)]^{1/2} \sum_i | I_{ih} - <I_h> | / \sum_h \sum_i I_{ih}$, in which $<I_h>$ is the mean intensity of symmetry-equivalent reflections h, I_{ih} is the intensity of a particular observation of h and N is the number of redundant observations of reflection h.[2]

[c] $CC_{1/2} = (<I^2> - <I>^2) / (<I^2> - <I>^2) + \sigma^2_\varepsilon$, in which σ^2_ε is the mean error within a half-dataset.[3]

[d] Root mean square deviation.

[e] calculated with PHENIX.[4]

[f] calculated with MOLPROBITY.[5]

[g] clashscore is the number of serious steric overlaps (> 0.4) per 1,000 atoms.[5]

Supplementary Table 4. Primers used for knockout generation and screening for knockout mutants.

Name	Sequence 5'→3'	Site
pamZ_118_IBS	AAAAAAGCTTATAATTATCCTTACATCTCCATAAAGTGCGCCCAGATAGGGTG	Knockout generation
pamZ_118_EBS1	CAGATTGTACAAATGTGGTGATAACAGATAAGTCCATAAATATAACTTAC-CTTTCTTTGT	Knockout generation
pamZ_118_EBS2	TGAACGCAAGTTTCTAATTTCGGTTAGATGTCGATAGAGGAAAGTGTCT	Knockout generation
pamZ_fw	GTGATGTGCCTCTCAATGCAG	Knockout screening
pamZ_rev	CCCAGCTTACTTGCCAGGTT	Knockout screening

Supplementary Table 5. Oligonucleotides used in this study.

Name	Sequence 5'→3'	Site
pET28a_pamZ_for	GGCCATATGGCsAGCATGCTGTATCTATTGGATAAATCGG	*Nhe*I
pET28a_pamZ_rev	CTCGAGTCTCGAGTTAATATTCAAATTCATAGGTTTTAAGTTTAAACC	*Xho*I

References

1. Liebschner, D. *et al.* Polder maps: Improving OMIT maps by excluding bulk solvent. *Acta Crystallogr. Sect. D Struct. Biol.* **73**, 148–157 (2017).
2. Word, J. M., Lovell, S. C., Richardson, J. S. & Richardson, D. C. Asparagine and glutamine: Using hydrogen atom contacts in the choice of side-chain amide orientation. *J. Mol. Biol.* **285**, 1735–1747 (1999).
3. Chen, V. B. *et al.* MolProbity: All-atom structure validation for macromolecular crystallography. *Acta Crystallogr. D Biol. Crystallogr.* **66**, 12–21 (2010).
4. Adams, P. D. *et al.* PHENIX: A comprehensive Python-based system for macromolecular structure solution. *Acta Crystallogr. D Biol. Crystallogr.* **66**, 213–221 (2010).
5. Williams, C. J. *et al.* MolProbity: More and better reference data for improved all-atom structure validation. *Protein Sci.* **27**, 293–315 (2018).
