# Supplementary material for: Molecular basis of antibiotic self-resistance in a bee larvae pathogen
Source: Nat Commun. 2022 Apr 29;13:2349. doi: 10.1038/s41467-022-29829-w (PMC9054821; doi:10.1038/s41467-022-29829-w)
Supplement: Supplementary file 3 — Reporting Summary [file 41467_2022_29829_MOESM3_ESM.pdf]

nature portfolio

Corresponding author(s): Prof. Dr. Roderich D. Süssmuth

Last updated by author(s): Mar 15, 2022

Reporting Summary

Nature Portfolio wishes to improve the reproducibility of the work that we publish. This form provides structure for consistency and transparency in reporting. For further information on Nature Portfolio policies, see our Editorial Policies and the Editorial Policy Checklist.

Statistics

For all statistical analyses, confirm that the following items are present in the figure legend, table legend, main text, or Methods section.

n/a	Confirmed	
☐	☒	The exact sample size (n) for each experimental group/condition, given as a discrete number and unit of measurement
☒	☐	A statement on whether measurements were taken from distinct samples or whether the same sample was measured repeatedly
☐	☒	The statistical test(s) used AND whether they are one- or two-sided<br>*Only common tests should be described solely by name; describe more complex techniques in the Methods section.*
☒	☐	A description of all covariates tested
☒	☐	A description of any assumptions or corrections, such as tests of normality and adjustment for multiple comparisons
☐	☒	A full description of the statistical parameters including central tendency (e.g. means) or other basic estimates (e.g. regression coefficient) AND variation (e.g. standard deviation) or associated estimates of uncertainty (e.g. confidence intervals)
☐	☒	For null hypothesis testing, the test statistic (e.g. F, t, r) with confidence intervals, effect sizes, degrees of freedom and P value noted<br>*Give P values as exact values whenever suitable.*
☒	☐	For Bayesian analysis, information on the choice of priors and Markov chain Monte Carlo settings
☒	☐	For hierarchical and complex designs, identification of the appropriate level for tests and full reporting of outcomes
☒	☐	Estimates of effect sizes (e.g. Cohen's d, Pearson's r), indicating how they were calculated

Our web collection on statistics for biologists contains articles on many of the points above.

Software and code

Policy information about availability of computer code

Data collection	XCalibur 2.2 (LTQ-Orbitrap XL hybrid ion trap-orbitrap, Thermo Fisher Scientific), MassHunter LC/MS Data Acquisition B.06.01 (6530 Accurate-Mass Quadrupole Time-of-Flight Q-TOF LC/MS, Agilent Technologies), TopSpin 3.5 (Bruker Avance III 700 MHz spectrometer, Bruker), Unicorn v5.20 (ÄKTApurifier10, GE Healthcare), Macromolecular crystallography beamline BL 14.2 at BESSY-II.
Data analysis	CorelDraw 2019, Adobe Illustrator CS4/CS6, ChemDraw v20.0 (PerkinElmer), TopSpin 3.5 (Bruker), NMRFAM-SPARKY (https://nmrfam.wisc.edu/nmrfam-sparky-distribution/), QualBrowser (XCalibur 2.2, Thermo Fisher Scientific), MassHunter Qualitative Analysis B.07.00 (Agilent Technologies), PyMOL v2.3.4 (Schrödinger Inc.), XDS, PHASER v2.8.1, Arp/wArp v8.0, PHENIX v1.16_3549, COOT v0.8.1, PHENIX-REDUCE v3.7.201124, APBS, PISA, DALI (http://ekhidna2.biocenter.helsinki.fi/dali/), Argus X1 v7.9.7 (Biostep), Clone Manager 7 (Sci Ed Software), GelDoc System v.0.2.14 (Intas Science Imaging Instruments GmbH), TargeTron (http://sigmaaldrich.com/crispr), QIAxcel ScreenGel v1.5.0.16 (Qiagen), BioTek Gen5 v1.10.8 (BioTek), GraphPad Prism v6.07 (GraphPad Software), Microsoft Excel (Microsoft Office Professional Plus 2019), antiSMASH 5.0 (https://antismash.secondarymetabolites.org/), protein BLAST (https://blast.ncbi.nlm.nih.gov/Blast.cgi), ClustalOmega (https://www.ebi.ac.uk/Tools/msa/clustalo/), Jalview v2.10.3b1 (https://www.jalview.org/), OligoAnalyzerTool (Integrated DNA Technologies).

For manuscripts utilizing custom algorithms or software that are central to the research but not yet described in published literature, software must be made available to editors and reviewers. We strongly encourage code deposition in a community repository (e.g. GitHub). See the Nature Portfolio guidelines for submitting code & software for further information.

Data

Policy information about availability of data

All manuscripts must include a data availability statement. This statement should provide the following information, where applicable:

- Accession codes, unique identifiers, or web links for publicly available datasets
- A description of any restrictions on data availability
- For clinical datasets or third party data, please ensure that the statement adheres to our policy

The MS data are shown in Figures 4 and 6, in Supplementary Figures S2-S17, S19-S23 and in Supplementary Table S1. In addition, the MS data that support the findings of this study have been deposited in Mass spectrometry Interactive Virtual Environment (MassIVE) with project identifier MSV000088695 [http://doi.org/10.25345/C5J86H]. The NMR data are shown in Figure 3, in Supplementary Figures S31-34 and in Supplementary Table S2. The chromatograms obtained from the size exclusion chromatography are depicted in Supplementary Figures S1 and S28. The crystallographic data collection and model refinement statistics have been listed in Supplementary Table S3. The coordinates and structure factors have been deposited in the Protein Data Bank under accession code 7B3A [http://doi.org/10.2210/pdb7B3A/pdb]. Diffraction images have been deposited at www.proteindiffraction.org. Additional crystal structures used in this study for molecular replacement and structural alignments are listed under the following accession codes: 3G3S [http://doi.org/10.2210/pdb3G3S/pdb], 1S3Z [http://doi.org/10.2210/pdb1S3Z/pdb], 1M4I [http://doi.org/10.2210/pdb1M4I/pdb]. The NCBI accession number of PamZ can be found here: WP_023484187 [https://www.ncbi.nlm.nih.gov/search/all/?term=WP_023484187%20].

Field-specific reporting

Please select the one below that is the best fit for your research. If you are not sure, read the appropriate sections before making your selection.

☒ Life sciences ☐ Behavioural & social sciences ☐ Ecological, evolutionary & environmental sciences

For a reference copy of the document with all sections, see nature.com/documents/nr-reporting-summary-flat.pdf

Life sciences study design

All studies must disclose on these points even when the disclosure is negative.

Sample size	Statistical methods were not used for the determination of sample sizes. The in vitro activation assay was repeated three times including negative controls for the MS experiment and performed once for the NMR experiment. Due to the clear evidence of the NMR spectra and additional validation by other experiments, the NMR experiment did not require replication. For the in vitro activation assay, the agar diffusion assay went along with the MS experiment and was repeated three times to visualize the conversion and inactivation. Compound extraction from the cell pellet of P. larvae including deletion mutant was conducted and analyzed twice independently. The in vivo activation assay including corresponding agar diffusion assay was repeated twice independently. All these experiments were determined qualitatively and the repeated experiments always gave identical results. The growth curves of P. larvae and deletion mutant were measured three times including three biological replicates additionally with three technical replicates for each biological replicate.
Data exclusions	Data were not excluded.
Replication	As described above, the MS experiments including the in vitro activation and corresponding agar diffusion assay were replicated three times. Both, the in vivo activation assay including corresponding agar diffusion assay as well as the compound extraction from the cell pellets were replicated twice independently. All replications always gave the same result. Since it is unusual to conduct replicates of X-ray and NMR measurements, both were only performed once, respectively. The acquisition of growth curves were replicated three times.
Randomization	In this study, randomization was not appropriate for the experimental setup since the biochemical assays required a rational approach to data collection and analysis in order to characterize the enzyme function.
Blinding	Due to the rational approach, blinding was not applicable to the biochemical assays, as the experiments did not involve human or animal subjects. But controls were always included in each biochemical assay.

Reporting for specific materials, systems and methods

We require information from authors about some types of materials, experimental systems and methods used in many studies. Here, indicate whether each material, system or method listed is relevant to your study. If you are not sure if a list item applies to your research, read the appropriate section before selecting a response.

Materials & experimental systems

n/a	Involved in the study
☒	☐ Antibodies
☒	☐ Eukaryotic cell lines
☒	☐ Palaeontology and archaeology
☒	☐ Animals and other organisms
☒	☐ Human research participants
☒	☐ Clinical data
☒	☐ Dual use research of concern

Methods

n/a	Involved in the study
☒	☐ ChIP-seq
☒	☐ Flow cytometry
☒	☐ MRI-based neuroimaging
